# The histone H2B Arg95 residue links the pheromone response pathway to rapamycin-induced G_1_ arrest in yeast

**DOI:** 10.1038/s41598-022-14053-9

**Published:** 2022-06-15

**Authors:** Abdallah Alhaj Sulaiman, Reem Ali, Mustapha Aouida, Balasubramanian Moovarkumudalvan, Dindial Ramotar

**Affiliations:** Division of Biological and Biomedical Sciences, College of Health and Life Sciences, Hamad Bin Khalifa University, Education City, Qatar Foundation, P.O. Box: 34110, Doha, Qatar

**Keywords:** Drug discovery, Cell biology, Cell signalling, Stress signalling

## Abstract

Rapamycin is an immunosuppressant used for treating many types of diseases such as kidney carcinomas. In yeast, rapamycin inhibits the TORC1 kinase signaling pathway causing rapid alteration in gene expression and ultimately cell cycle arrest in G_1_ through mechanisms that are not fully understood. Herein, we screened a histone mutant collection and report that one of the mutants, H2B R95A, is strikingly resistant to rapamycin due to a defective cell cycle arrest. We show that the H2B R95A causes defects in the expression of a subset of genes of the pheromone pathway required for α factor-induced G_1_ arrest. The expression of the *STE5* gene and its encoded scaffold protein Ste5, required for the sequential activation of the MAPKs of the pheromone pathway, is greatly reduced in the H2B R95A mutant. Similar to the H2B R95A mutant, cells devoid of Ste5 are also resistant to rapamycin. Rapamycin-induced G_1_ arrest does not involve detectable phosphorylation of the MAPKs, Kss1, and Fus3, as reported for α factor-induced G_1_ arrest. However, we observed a sharp induction of the G_1_ cyclin Cln2 (~ 3- to 4-fold) in the *ste5Δ* mutant within 30 min of exposure to rapamycin. Our data provide a new insight whereby rapamycin signaling via the Torc1 kinase may exploit the pheromone pathway to arrest cells in the G_1_ phase.

## Introduction

Rapamycin is an immunosuppressant used for treating diseases such as kidney carcinomas^1–3^. In *Saccharomyces cerevisiae*, rapamycin binds to the peptidyl-prolyl isomerase Fpr1 leading to the inhibition of the Target of Rapamycin (TOR) kinase that forms the TOR Complex 1 (Torc1)^4,5^. Torc1 consists of either the Tor1 or the Tor2 kinase, a putative scaffold protein Kog1, Lst8, and Tco89^4,5^. Torc1 controls growth in response to nutrients by regulating translation, transcription, ribosome biogenesis, nutrient transport, autophagy, and cell cycle^4,6–8^. The inhibition of Torc1 by rapamycin mimics nutrient starvation and causes an array of physiological changes that include global changes in gene expression, phosphorylation/dephosphorylation of various factors, decreased cell growth, degradation of proteins, and ultimately cell cycle arrest in the G_1_ phase^4,9–11^. The exact molecular mechanism that leads to G_1_ arrest following rapamycin treatment in yeast is a complex process that has not been fully delineated, although it is believed to be indirectly derived from a combination of events that include (i) inhibition of protein synthesis due to the degradation of the protein translational initiation factors eIF4G1 and eIF4G2^10,12–14^, (ii) reduced expression of genes encoding ribosomal proteins^8^, (iii) the subsequent transcriptional downregulation of the cyclin genes involved in the transition of cells from the G_1_ to the S phase^10^, (vi) the specific phosphorylation at the C-terminal domain of RNA polymerase II^15^, and (v) the phosphorylation and stabilization of the Sic1 inhibitor protein that inhibits the B-type cyclin-dependent protein kinase^16–18^.

We have previously shown that the peptidyl-prolyl isomerase Rrd1 is required to mediate cellular response to rapamycin by altering gene expression^6,7,19,20^. Rrd1 is tightly bound to chromatin and interacts with RNA polymerase II (RNAPII)^6,20^. It can isomerize the C-terminal domain of the large subunit of RNAPII and this may promote the redistribution of the polymerase along the genome in response to rapamycin^6,20,21^. Rrd1 has been shown to stimulate the association of RNAPII with the coding region of inducible genes besides those activated by rapamycin, but not with constitutively expressed genes^6,21^. This Rrd1-dependent recruitment of RNAPII is associated with nucleosomal disassembly and transcription activation^6,20,21^. From these combined studies, it seems logical that the global gene expression pattern that ensues upon exposure to rapamycin must be governed by changes at the level of the chromatin structure. Indeed, it has been shown that a histone mutant H3 K56A causes yeast cells to display sensitivity to rapamycin, however, this mutation also sensitizes cells to a variety of DNA damaging agents, suggesting that the H3 K56A mutation has a broader role rather than a specific function towards rapamycin stress^22,23^. Here, we hypothesized that specific histone residue(s) might be involved in facilitating gene expression such that cells mount a response to rapamycin. We, therefore, searched a collection of 442 histone mutants^24^ for ones that failed to respond to rapamycin in an attempt to identify molecular pathways critical for rapamycin response at the level of chromatin. We report the identification of nine histone mutants, eight of which showed varying resistance to rapamycin, as compared to the other mutants and the wild type (WT). Of these mutants, H2B R95A displayed striking resistance to rapamycin, which correlated with an inability to undergo cell cycle arrest. Further analysis revealed that the H2B R95A was significantly defective in the expression of at least 26 genes that belong to the pheromone response pathway that is required for α factor-induced G_1_ arrest. This mutant weakly expressed the *STE5* gene and its encoding scaffold protein Ste5 is required for the sequential activation of the MAPKs of the pheromone pathway. Cells lacking Ste5 exhibited nearly the same level of resistance to rapamycin as the H2B R95A mutant, suggesting that the rapamycin-resistant phenotype of this mutant is restricted to the pheromone response pathway. We show that rapamycin-induced G_1_ arrest is independent of the phosphorylation of the MAPKs, Kss1 and Fus3, required for α factor-induced G_1_ arrest. However, we unexpectedly observed a sharp induction of the G_1_ cyclin Cln2 in the *ste5Δ* mutant within 30 min of exposure to rapamycin, as compared to the WT. We provide some insights into the possible roles of the induced Cln2 level in the *ste5Δ* mutant, which might serve to promote cell proliferation in the presence of rapamycin.

## Materials and methods

### Yeast strains, growth media, plasmid, and drugs

The yeast WT strains and the isogenic histone mutants used in this work (Supplementary Table S1) were from the SHIMA library, kindly provided by Dr. Ali Shilatifard (Kansas, USA)^24^. All other strains used in this study (Supplementary Table S1) were the WT BY4741 and the indicated isogenic mutants derived from the nonessential haploid mutant (D.R. laboratory resource) and the TAP-tagged collections provided by Dr. Hugo Wurtele (Montreal, Canada). Epitope-tagging of strains at the endogenous gene locus was performed as previously described^25^. Cells were grown at 30 °C for 24 h in either Yeast Peptone Dextrose (YPD, FORMEDIUM CCM0105) or SD minimal media. The single-copy plasmid pSTE5-GFP carrying the entire *STE5* gene under its promoter and tagged with GFP was kindly provided by Dr. Peter M. Pryciak (University of Massachusetts medical school, Worcester, MA, USA). The plasmid pSTE5-Myc was constructed by gap repair using pTW438 as the backbone and as previously decsribed^26^. All chemical reagents including rapamycin, methyl methanesulfonate, 4-nitroquinoline-1-oxide, bleomycin and sodium arsenite were purchased from Sigma, St Louis, USA.

### Spotting test

The strains were grown in YPD media at 30 °C for 24 h. The OD600_nm_ was adjusted to 1.0 and serial dilutions of 1:10, 1:50, 1:100, 1:500, and 1:1000 were prepared using 96-well plates^6^. Four microliters of each dilution were spotted onto YPD solid media without and with 2.0 ng/ml of rapamycin. The plates were incubated at 30 °C and photographed after 48 h using (Image Lab Touch Software, BioRad).

### RNA extraction and RT-PCR

Yeast cultures were grown overnight, then collected the following day by centrifugation at 3220×*g* for 5 min. The pellets were washed once with sterile water, then processed for RNA extraction using RiboPure™ yeast RNA purification kit as per the manufacturer protocol (Qiagen). RNA concentration and purity were checked by Nanodrop 2000. cDNA was prepared from the total RNA (0.5 µg) using high-capacity cDNA reverse transcription kit according to the manufacturer protocol (Thermofisher Scientific). Samples were run on Quanti Studio 6 Flex qPCR machine using PowerUp SYBR Green Master Mix (Thermofisher Scientific), *ACT1* gene was used as the control for quantification of *STE5* gene expression, and the primers used were as follow: ACT1-F, 5ʹ-TGGGTATCCAAGCACATCAA; ACT1-R, 5ʹ-TGATAAACCCGCTGAA CACA; STE5-F, 5ʹ-CGTCCGGAGCAAACTCTATC; and STE5-R, 5ʹ-ATGACCTTAACAGC GGCAAC. Data normalizations were performed using the 2(-Delta Delta C(T)) method.

### Protein extraction and immunoblotting

Exponentially growing yeast strains were pelleted at 3220×*g* for 5 min. The supernatants were discarded and the pellets were resuspended in 200 µl of 20% trichloroacetic acid (TCA)^20^. Then transferred to 1.5 ml tubes containing 300 µl of yeast cell extraction glass beads (0.5 mm diameter, BioSpec Cat. No. 11079105). Cells were lysed using a bead mill homogenizer (BeadMill 4, FisherScientific) at 5 m/s for 5 s and repeated 10 times. The supernatants were transferred to new 1.5 ml Eppendorfs, then the beads were topped up with 150 µl of 5% TCA and re-homogenized in the bead mill homogenizer at 5 m/sec for 5 s and repeated 10 times. The supernatants were combined with the previous 200 µl from the initial extracts. The collected TCA extracts were centrifuged for 10 min at 10,000 rpm (or 9391×*g*) using an Eppendorf microcentrifuge at 4 °C. The supernatants were discarded and the remaining pellets were washed once in 70% ethanol to remove residual TCA. The pellets were resuspended in 100 µl of 1 × SDS loading buffer. The extracts will turn yellow colour because of the pH change. The pH was restored by adding 5 µl of 1 M Tris base solution that was not pH adjusted. Protein samples were loaded on SDS-PAGE gels. Separated proteins were transferred to 0.22 µm nitrocellulose membranes, presoaked in transfer buffer (48 mM TRIS, 390 mM Glycine, 20% methanol, and 1% SDS) using the Trans-Blot Turbo transfer system at 25 V for 30 min. Membranes were blocked in 5% non-fat milk in TBS-Tween (10 mM Tris–HCl pH 7.5, 15 mM NaCl, 1.2 mM EDTA, and 0.1% Tween 20) for 1 h, then incubated with the indicated antibodies overnight at 4 °C. Antibodies used in the study were anti-Phospho-p44/42 MAPK (Erk1/2) Thr202 /Tyr204 (rabbit mAB 4370, Cell Signaling) generously provided by Dr. Essam Abdelalim (Qatar Biomedical Research Institute, Qatar), anti-GFP IgG (Roche antibodies), and anti-PAP (Sigma-Aldrich, P1291, St. Louis, MO, USA). The following day membranes were washed with TBS-Tween three times for 5 min each then probed with HRP-Affinity pure (H + L) secondary antibodies 1:5000 (Jackson Immunoresearch) for 1 h at room temperature. Membranes were visualized using Pierce™ ECL Western Blotting Substrate (Thermofisher Scientific).

### FLAG-H2B purification by anti-FLAG affinity gel

Cell pellets from exponentially growing cells in YPD medium were resuspended in 4 volumes of native lysis PBS buffer containing (5 mM EGTA, 5 mM EDTA, 0.5% Triton X-100, 0.5% Nonidet-P40, 10% glycerol, 50 mM sodium fluoride, 10 mM β-glycerophosphate, 5 mM sodium pyrophosphate, 5 mM sodium orthovanadate, 1 × protease inhibitor cocktail, 2 mM PMSF) and lyzed by mechanical shearing using acid-washed glass beads as previously described^27^. After separation from glass beads and centrifugation at 16,000×*g* for 10 min at 4 °C, the supernatant was incubated with anti-FLAG M2 affinity gel (Sigma) for 3 h at 4 °C, and then washed with native lysis PBS buffer and TBS (10 mM Tris–HCl pH 7.5 and 15 mM NaCl), three times each. Immunoprecipitates were eluted by elution buffer (50 mM Tris–HCl, pH 7.5, 10 mM EDTA, 1% SDS) for 20 min at 42 °C^27^.

### Mass spectrometry

Polypeptide bands were excised from the silver-stained gels and subjected to micro-capillary LC/MS/MS analysis mass spectrometry for identification and or modifications (Taplin Mass Spectrometry Facility, Harvard Medical School, Boston, MA, USA).

### Cell cycle analysis

Overnight cultures were sub-cultured for 3–4 h, samples were taken as untreated controls, and then the cells were treated with either α-factor or rapamycin (see figure legends). Following the treatment, the cells were washed and placed in fresh media to allow for recovery. During the recovery phase, samples were taken at the indicated times for cell cycle analysis as previously described^28^.

### Hot phenol extraction to prepare yeast RNA for Microarray using Affymetrix arrays

Exponential cultures (50 ml of OD_600_ = 0.7) were either untreated or treated with rapamycin (200 ng/ml for 30 min), cells harvested (4000 rpm (3220×*g*) for 2 min), washed once by resuspending in 6 ml of ice-cold sterile H_2_O, immersed into liquid nitrogen to snap freeze the cell pellet followed by immediate storage at − 80 °C. The next day, the cell pellet was thawed on ice, and total RNA was extracted using the hot phenol extraction protocol^29^.

### Microarray data analysis

Microarray analysis was performed using Affymetrix GeneChip^®^ Microarray at Genome Québec Innovation Centre and McGill University. The Affymetrix raw data “.CEL” files were imported into the FlexArray (version 1.6.3) followed by execution of robust multi-array average (RMA), which performs the background correction and quantile normalization. Principal components analysis (PCA) was used as a tool to perform quality control for the data. For subsequent statistical analysis, we used the cyber-T (Baldi and Long), Bayesian framework implemented in Flex Array. Genes up-regulated (≥ twofold change) and down-regulated (≤ 0.5 fold change) with high statistical significance (p-value ≥ 0.01) were exported as text files (“.TXT”) for further analysis. Volcano plots of the differentially expressed genes were generated using − Log_10_ (p-value) and Log_2_ (Fold change). Venny 2.0.2 (Computational Genomic Services, CSIC) was employed for comparing gene lists and drawing Venn’s diagram. Functional annotation clustering of the differentially expressed genes related to biological pathways was then performed using Gene Ontology (GO) term enrichment analysis and KEGG pathway mapping through DAVID Bioinformatics Resources 6.7 (http://david.abcc.ncifcrf.gov) with ease score = 0.01 and similarity threshold = 0.50. GO terms significantly represented among differentially expressed genes were then listed with their corresponding p-value and FDR in Supplementary Table II. False discovery rates were controlled using the Benjamini Hochberg method. GO annotation of genes was obtained from the Affymetrix database. The datasets analyzed in this study are available in the GEO DataSets repository (see availability of data below).

### Consent for publication

All authors gave their consent for publication.

## Results

### Identification of histone mutants with altered cellular response to rapamycin

**Figure 1:**
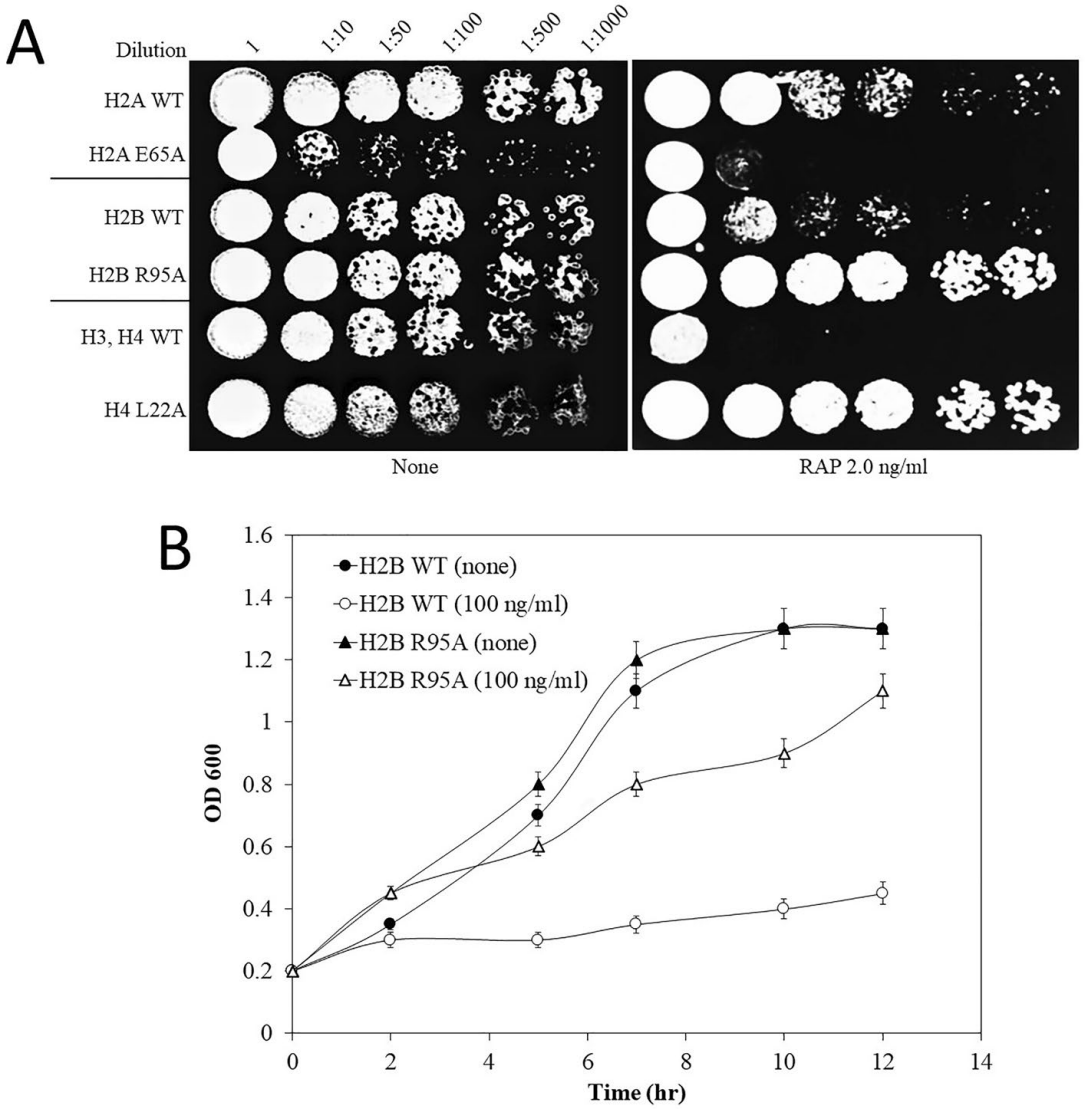
Comparison of the rapamycin sensitivity or resistance of the selected histone mutants with the respective WT. (**A**) The selected histone mutants and the respective wild type (WT) strains H2A WT, H2B WT, or H3/H4 WT were grown overnight in a 96-well plate and the next day, the optical density (OD 600) was adjusted to 1.0 then serially diluted followed by spotting of 4 µl of the cells onto YPD solid media without and with rapamycin (RAP 2.0 ng/ml). Plates were incubated for 48 h at 30 °C. The data shown are representative of several independent experiments. (**B**) Growth of the H2B WT and the mutant H2B R95A in the absence and presence of rapamycin (100 ng/ml). The overnight cultures were inoculated at low OD 600 (0.2) in 96-well plates in a final volume of 200 µl. The OD 600 of the cells was measured at the indicated time points. The results shown are the averages of three independent experiments and the error bars indicate the standard deviation.

A collection of 442 yeast strains expressing mutated versions of the core histones were created by replacing each amino acid residue of H2A, H2B, H3, and H4 with alanine. The individual variant carried by a single copy plasmid was introduced into a modified parental strain deleted for both copies of the respective endogenous histone genes^24^. We screened the entire histone mutant collection to identify those that were either sensitive or resistant to rapamycin using spot test analysis. This screen identified a single mutant H2A E65A showing sensitivity to rapamycin, as compared to the WT (Fig. 1A). However, this H2A E65A mutant also showed sensitivity to several other agents such as the environmental toxic metalloid sodium arsenite and the DNA damaging agents methyl methanesulfonate (MMS) (Supplementary Figs. S1 and S2), suggesting that H2A E65A might affect multiple cellular functions. In addition, the screen identified eight mutants displaying variable degrees of resistance to rapamycin when compared to the strains expressing WT histones (Fig. 1A, Supplementary Fig. S3). The eight rapamycin-resistant mutants showed parental resistance towards arsenite (Supplementary Fig. S1). Seven of these rapamycin resistant mutants (H2B R95A; H3 G12A; H3 V46A; H3 E94A; H4 R19A; H4 L22A; and H4 R35A) were not detected as mutants exhibiting sensitivity or resistance to the DNA damaging agents MMS and bleomycin (BLM), except for H4 R36A showing sensitivity to both of these drugs (Supplementary Fig. S2 showing only the MMS and BLM sensitive mutants). The data suggest that these seven histone residues may be specifically responding to rapamycin.

The histone mutants that were resistant to rapamycin and showed WT responses to arsenite and the DNA damaging agents were found within histones H2B, H3, and H4, and none was recovered from the set of H2A alleles (Fig. 1, Supplementary Fig. S3). Only one mutant, H2B R95A, was recovered from the histone H2B collection that exhibited prominent resistance to rapamycin amongst all the histone mutants when tested semi-quantitatively by the spot test analysis (Fig. 1A, Supplementary Fig. S3). This H2B R95A mutant was independently checked for growth rate in liquid culture by assessing for cell density at OD 600 throughout 12 h in the absence and presence of rapamycin. The H2B WT and H2B R95A mutant cells proliferated at the same rate in the absence of rapamycin (Fig. 1B). However, in the presence of rapamycin growth of the H2B WT was significantly impaired by 2.5 h, while the H2B R95A mutant continued to grow (Fig. 1B). This latter analysis is consistent with the H2B R95A mutant being defective in a process that attenuates cell growth upon exposure to rapamycin.

In the case of histone H3, only three rapamycin-resistant mutants H3 G12A, H3 V46A, and H3 E94A were recovered, although these were not as resistant to rapamycin as H2B R95A (Supplementary Fig. S3). The H4 mutants (R19A, L22A, R35A, and R36A) were located primarily in the N-terminal part of the protein. The H4 L22A mutant was more resistant to rapamycin as compared to H4 R19A, H4 R35A, and H4 R36A and less resistant as the H2B R95A mutant when challenged with higher concentration of rapamycin (Supplementary Fig. S3). It is noteworthy that the N-terminal tail of H4 makes contact with the H2A-H2B dimer, and plays a major role in many processes including replication-coupled chromatin assembly and gene expression^30^. Collectively, it appears that a narrow set of histone residues can specifically cause resistance to rapamycin when replaced with alanine. Since the histone H2B R95A mutant was strikingly more resistant to rapamycin (Supplementary Fig. S3) and did not display sensitivity or resistance to other agents^23^, we focused on unraveling its role in response to rapamycin.

### Microarray analysis reveals that H2B R95A downregulates several genes in the pheromone pathway

**Figure 2:**
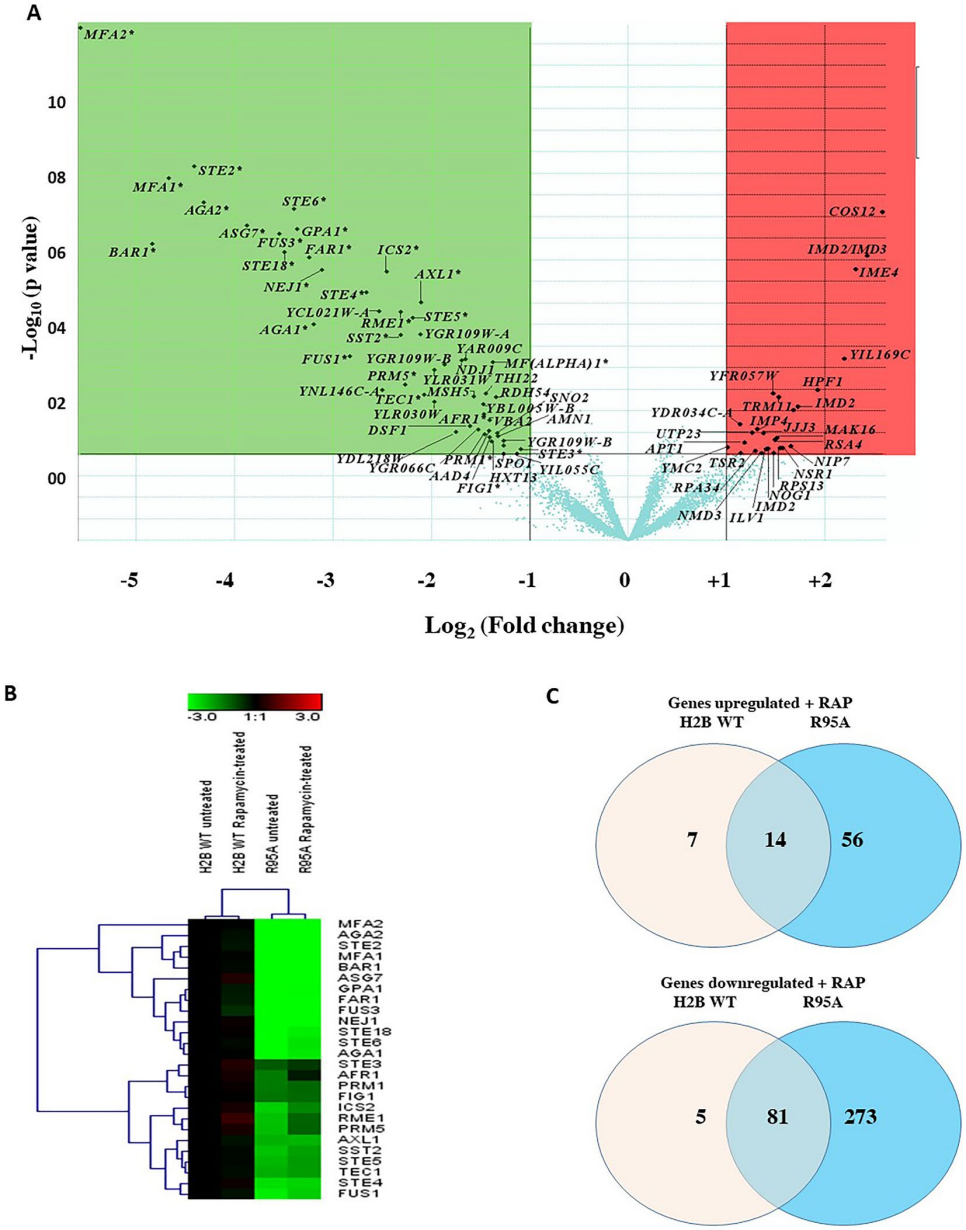
Effects of the H2B R95A mutation on gene expression in the absence and presence of rapamycin. (**A**) Volcano plot showing the genes differentially regulated in the H2B R95A with respect to H2B WT cells. The volcano plot was generated by plotting the negative log_10_ p-value (as the y-axis) of the cyber T-test against the log_2_ fold change (as the x-axis). Genes indicated with an asterisk (*) belong to the pheromone response pathway in yeast. (**B)** Heat map visualization obtained by hierarchical clustering of the pheromone pathway genes downregulated in H2B R95A mutant under no treatment or rapamycin treatment (200 ng/ml for 30 min) as compared to H2B WT. This analysis is based on a subset of n = 26 genes (rows) of the pheromone response pathway in yeast that were found to be differentially expressed in H2B R95A mutant. The color-ratio bar at the top indicates the intensity of gene up-regulation (red), down-regulation (green), and no change (black). (**C**) Venn diagram showing the response of histone H2B WT and H2B R95A to rapamycin treatment. Venn diagram created using the genes upregulated or downregulated in histone H2B WT and H2B R95A under rapamycin treatment.

To obtain molecular insights into how H2B R95A brings about rapamycin resistance, we compared the gene expression pattern of the H2B R95A mutant with the H2B WT under normal growth conditions and when the cells were treated with rapamycin (200 ng/ml for 30 min). A volcano plot of genes differentially expressed under normal conditions revealed that 25 genes, mainly belonging to ribosome biogenesis and RNA processing, were upregulated (p-value of ≤ 0.01) in the H2B R95A mutant as compared to the WT (Fig. 2A, shown in red) (see GEO DataSets repository, accession number GSE201413). More than 50 genes were downregulated in the mutant as compared to the WT (Fig. 2A, shown in green), and of these, 26 belong to the pheromone response pathway^31^. These results suggest that the Arg95 residue of H2B plays a predominant role to ensure proper regulation of the pheromone pathway genes, consistent with the observation of Dai et al. that this mutant has a defect in mating^32^.

Rapamycin did not affect the expression profile of these pheromone pathway genes in the H2B WT or H2B R95A mutant using two-dimensional hierarchical clustering (Supplementary Fig. S4). This analysis grouped the non-treated and rapamycin-treated WT and the H2B R95A mutant in distinct clusters, implying that rapamycin did not affect the expression profile of these pheromone pathway genes (Fig. 2B).

We further analyzed the data by comparing genes that were differentially expressed between the WT and the H2B R95A mutant following rapamycin treatment using Venn diagrams (Fig. 2C). The analysis showed that rapamycin treatment caused up-regulation of 70 genes in the H2B R95A mutant and 21 in the H2B WT, with 14 being common to both strains (Fig. 2C). In contrast, a considerable number of genes (354) were significantly down-regulated in the H2B R95A mutant and 86 in the H2B WT with 81 being common to both strains (Fig. 2C). Almost all the differentially expressed genes in the H2B R95A mutant were enriched in the same GO term as the H2B WT and related, e.g., to ribosome biogenesis, RNA processing, RNA maturation, and export from the nucleus (Supplementary Table S2). This analysis suggests that the H2B R95A mutant is still capable of altering the expression of a subset of genes similar to the H2B WT in response to rapamycin.

### H2B R95A mutant drastically reduces the expression of the *STE5* gene and its encoded protein

**Figure 3:**
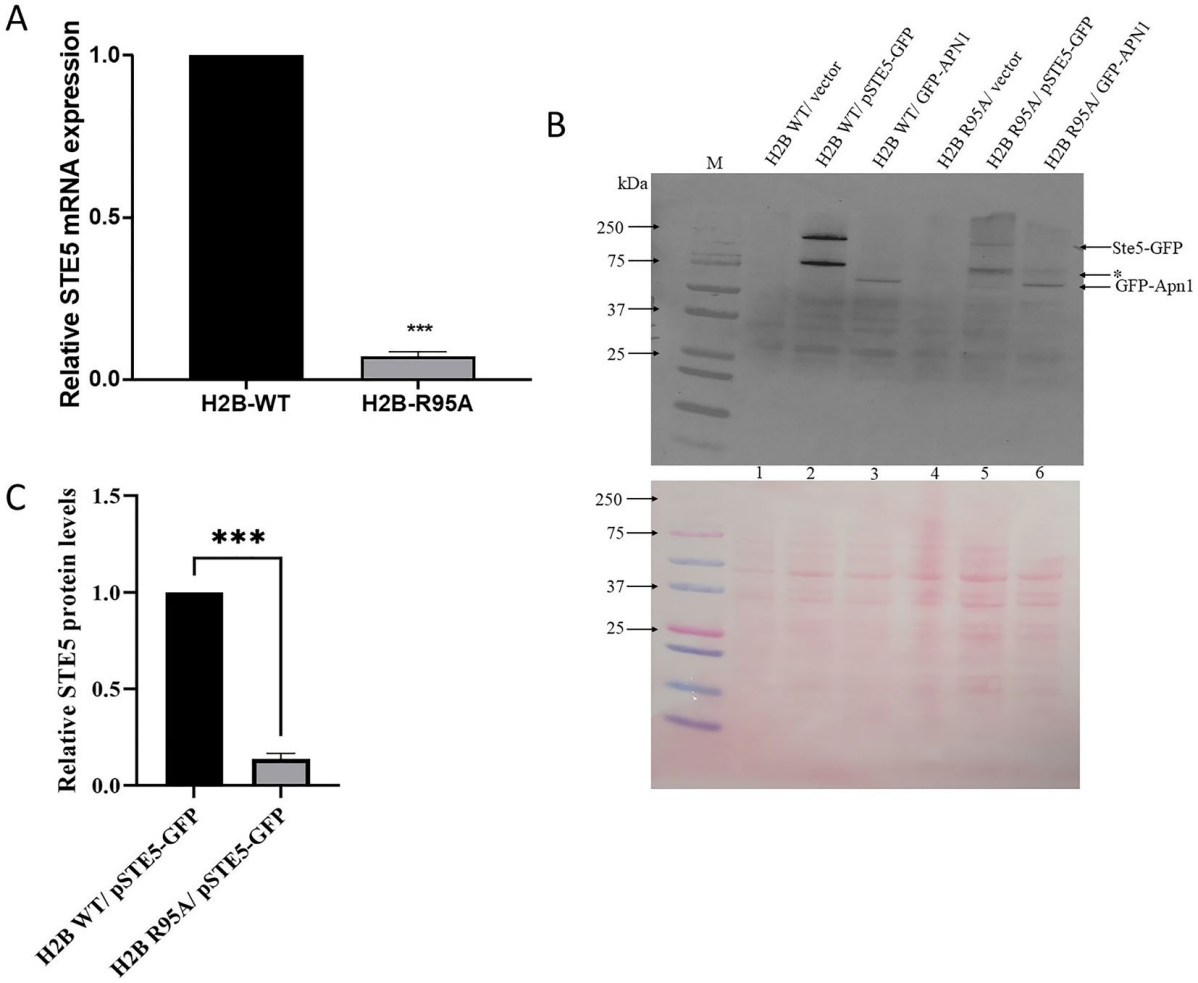
H2B R95A mutant drastically reduces the expression of the *STE5* gene and its encoded protein. (**A**) Relative mRNA expression of *STE5* gene by qPCR analysis. Total RNA was isolated from the H2B WT and H2B R95A strains and 0.5 µg was used for reverse transcribed using the yeast RiboPure kit. The cDNAs were subjected to qPCR analysis with indicated primers (see “Materials and methods”). The *ACT1* gene was used as a control. The data are representative of four biological replicates and analyzed by student *t*-test. ***Is equivalent to P-value < 0.001. (**B**) Immunoblot analysis of Ste5-GFP and GFP-Apn1 in the H2B WT and H2B R95A. Plasmids expressing either Ste5-GFP or GFP-Apn1 were introduced into the H2B WT and H2B R95A. Total cell extracts were prepared from these strains with the trichloroacetic acid method (see “Materials and methods”) and process for immunoblot analysis. The blot was probed with anti-GFP antibodies (upper panel). The lower panel was stained with Ponceau to monitor for equal protein loading. M, prestained protein markers in kDa. Arrows indicate the position of the GFP-tagged proteins, and the asterisk indicates a fragmented species of Ste5-GFP. (**C**) The expression level of the full-length Ste5-GFP in the H2B R95A mutant was quantified from panel (**B**) and expressed relative to the level detected in the H2B WT strain.

In the pheromone response pathway, the scaffold protein Ste5 and its associated kinases Ste11, Ste7, Fus3, and Kss1 are recruited to the α-factor receptor Ste2 located at the plasma membrane when cells are exposed to α-factor. This brings the MAPKKK Ste11 in proximity with another membrane-bound complex containing the Ste20 kinase^31,33,34^. Ste20 phosphorylates Ste11 which then activates the MAPKK Ste7, which in turn activates the MAPKs Kss1 and Fus3^31,33,34^. The activated Fus3 kinase translocates to the nucleus leading to the downregulation of the G_1_ cyclins including Cln1 and Cln2 causing cell cycle arrest^35,36^. Since Ste5 is a crucial component that allows the assembly and communication between the kinases, and mutants deleted for the *STE5* gene cannot transmit a signal along the pheromone pathway to activate the MAPKs^34^, we checked whether *STE5* gene expression is indeed downregulated by H2B R95A mutant as revealed by the microarray data (Fig. 2). Total RNA was isolated from H2B WT and the H2B R95A mutant and the cDNAs derived from reverse transcriptase were used to quantify *STE5* gene expression by quantitative PCR and normalized against the *ACT1* gene. The analysis revealed that the *STE5* gene was dramatically downregulated in the H2B R95A mutant as compared to the H2B WT, consistent with the microarray data (Figs. 2A, 3A, respectively).

We next checked whether the Ste5 protein would also be similarly downregulated in the H2B R95A mutant. To do this, we introduced a single-copy plasmid pSTE5-GFP expressing Ste5 from its promoter, as a GFP fusion protein, into the H2B WT and H2B R95A strains and monitored Ste5-GFP expression level by immunoblot analysis with anti-GFP antibodies, as no commercial Ste5 antibody is available^37^. The Ste5-GFP was highly expressed in the H2B WT strain, but only weakly expressed in the H2B R95A mutant (Fig. 3B, lane 2 vs. 5, and Fig. 3C showing the quantification of the expressed full-length Ste5-GFP in the H2B R95A mutant relative to the H2B WT) (for the full immunoblot see Supplementary Fig. S5). Under the extraction conditions, some of the native Ste5-GFP protein was apparently proteolytically processed to a lower molecular weight isoform of ~ 80 kDa, which is not present in the vector control strains H2B WT and H2B R95A (Fig. 3B, shown by an asterisk). A control protein GFP-Apn1 driven from the galactose-inducible promoter *GAL1* under the non-induced condition was expressed at the same level in both the H2B WT and H2B R95A strains (Fig. 3B lane 3 and 6, respectively). These findings support the notion that the pheromone response pathway is disrupted in the H2B R95A mutant.

### *ste5* null mutant displays resistance to rapamycin

**Figure 4:**
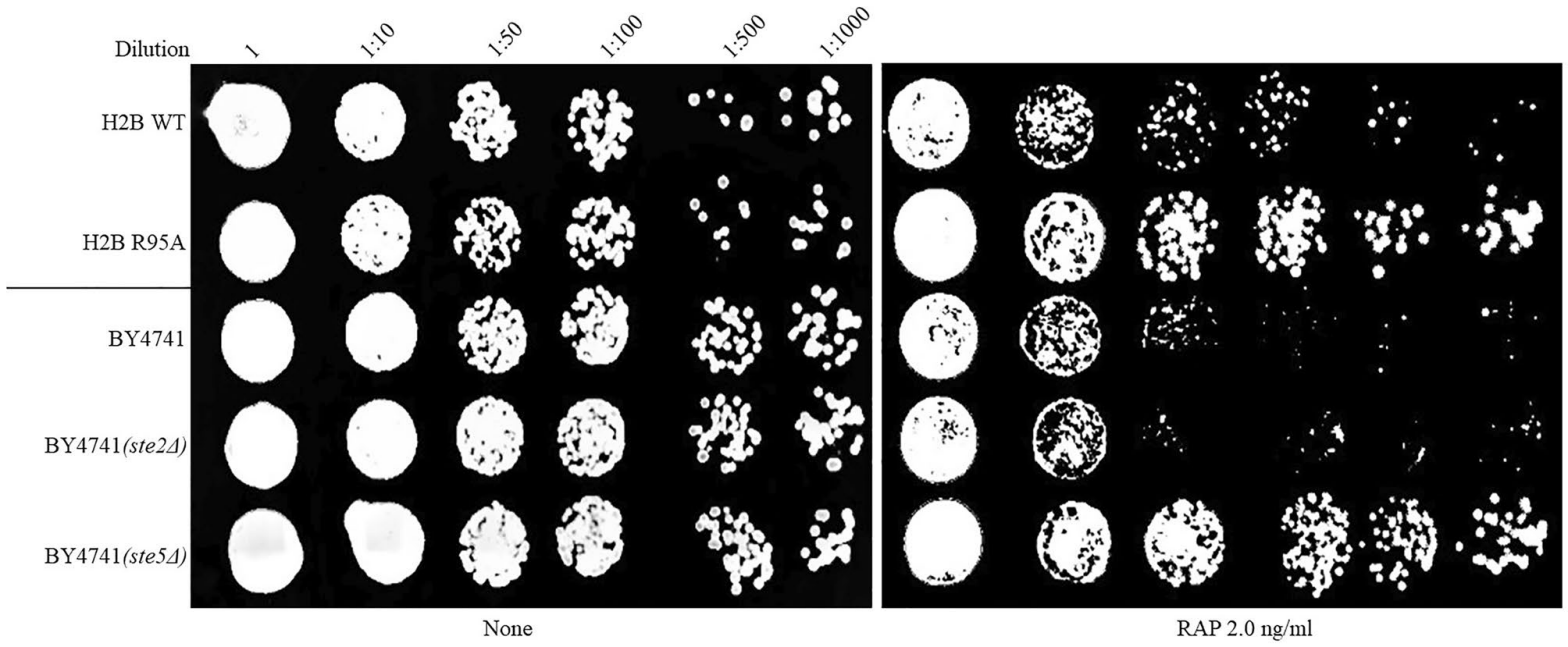
Ste5 is required to mediate cellular response to rapamycin. Spot test analysis of the indicated strains was performed as described in Fig. 1A. The *ste2Δ* and *ste5Δ* null mutants are isogenic to the WT strain BY4741. The experiment was repeated at least three times.

To determine whether Ste5 is involved in controlling the cellular response to rapamycin, we used a different and most common parental background strain BY4741 and its isogenic *ste5Δ* mutant and tested for rapamycin resistance using spot test analysis. As shown in Fig. 4, the *ste5Δ* mutant displayed resistance to rapamycin as compared to the parent BY4741, suggesting that Ste5 is required to channel the rapamycin signal to trigger growth arrest. Similar results were obtained when the *STE5* gene was deleted from the W303 parental background (Supplementary Table S1). In contrast, the *ste2Δ* mutant lacking the transmembrane α-factor receptor protein Ste2 did not show resistance to rapamycin when compared to the WT (Fig. 4). Based on this observation, it would appear that Ste2 is not required for the recruitment of Ste5 and its associated proteins to the plasma membrane to signal the response by rapamycin.

### Rapamycin induces G_1_ arrest in the H2B WT, but not in the H2B R95A mutant

**Figure 5:**
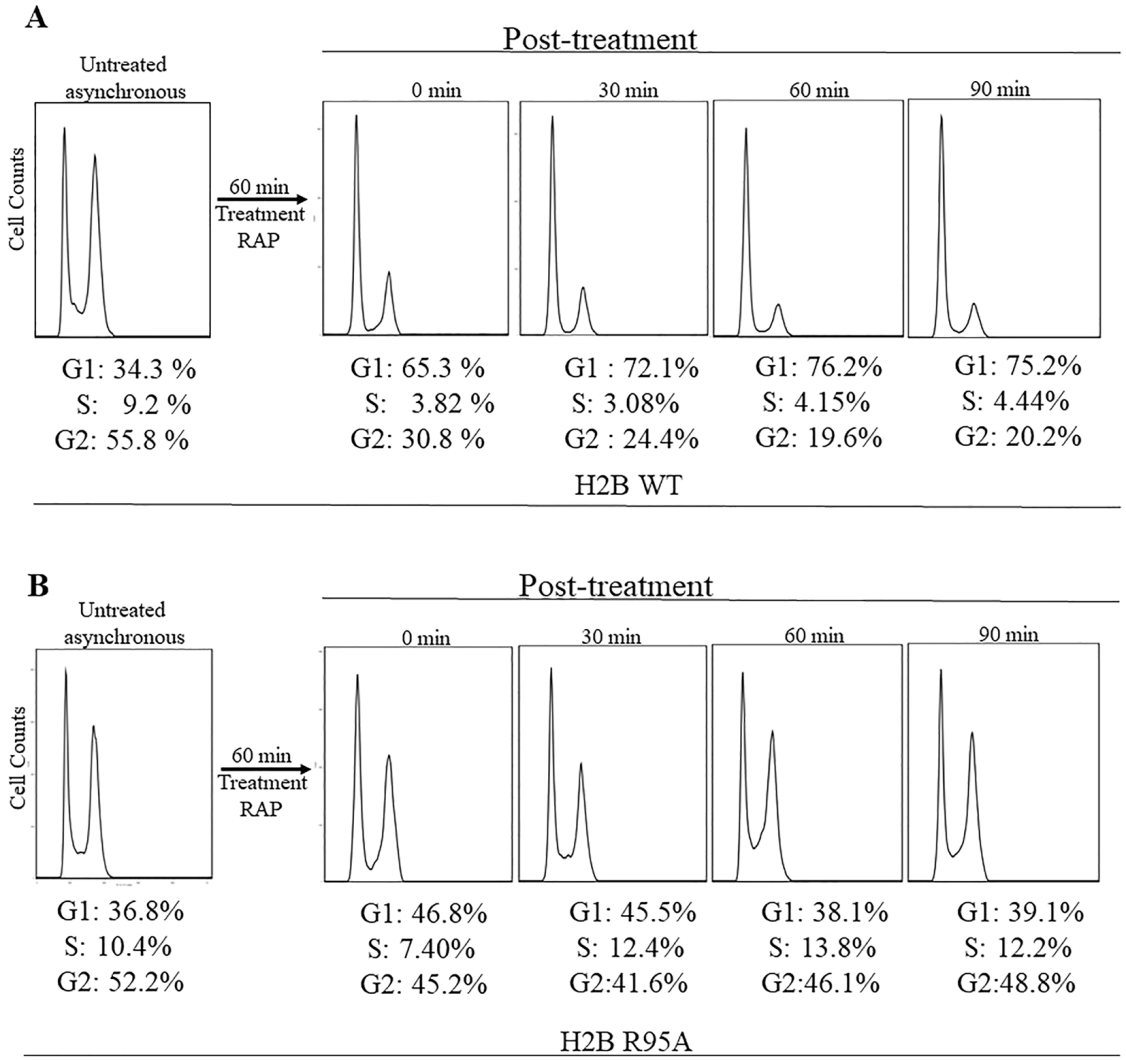
Rapamycin induces G_1_ arrest in the H2B WT, but not in the H2B R95A mutant. (**A**,**B**) Briefly, overnight cells were sub-cultured for 3 h and samples were taken for asynchronous growth followed by treatment without and with rapamycin (RAP; 200 ng/ml for 30 min). Cells were washed free of rapamycin, incubated in fresh media, and samples were taken at time zero after the treatment, followed by sampling at 30, 60, and 90 min. Samples were processed using FACS analysis and the image treated by the Flowjo software. The results are representative of two independent analyses.

When WT cells are challenged with α-factor, the signal is transmitted along the pheromone pathway leading to cell cycle arrest in the G_1_ phase^31^. If rapamycin uses this same pathway to signal G_1_ arrest^10^, then it is anticipated that this arrest would be disrupted in the H2B R95A mutant. Exponentially growing asynchronous H2B WT cells were treated with rapamycin (200 ng/ml for 60 min), then washed to remove the drug, followed by post-treatment recovery in fresh media, and the samples taken at the indicated time were processed by Fluorescence-Activated Cell Sorting (FACS) analysis (Fig. 5). The H2B WT cells were rapidly arrested in the G_1_ phase following the rapamycin treatment (Fig. 5A, second panel) and remained in the G_1_ phase during the duration of the sampling (90 min) in the fresh media without rapamycin (Fig. 5A, third, fourth, and fifth panels). In contrast, the asynchronous population of the H2B R95A mutant cells failed to arrest in the G_1_ phase following rapamycin treatment (Fig. 5B vs. A). This observation is consistent with the H2B R95A mutant ability to grow in the presence of rapamycin (Fig. 1). We interpret these findings to indicate that rapamycin may transmit a signal through the pheromone response pathway to arrest the cell cycle in the G_1_ phase.

In the control experiment, exponentially growing cultures were treated with α-factor for 3 h, washed free of the α-factor, and the cell cycle release was monitored over time post-treatment in fresh media using FACS. The asynchronous cell populations for both the H2B WT and the H2B R95A mutant showed a similar proportion of cells in the G_1_ and G_2_ phases (Supplementary Fig. S6A). As expected, following α-factor treatment, the H2B WT cells were arrested in the G_1_ phase and re-entered the cell cycle upon removal of the α-factor (Supplementary Fig. S6A). In contrast, the H2B R95A mutant failed to arrest in the G_1_ phase, even after prolonged exposure to the α-factor, and continued to progress normally in the cell cycle (Supplementary Fig. S6B). This finding confirms that the H2B R95A mutant is indeed defective in the pheromone response pathway.

### Fus3, a downstream component of the pheromone pathway, is expressed in the H2B WT, but not detected in the H2B R95A mutant

**Figure 6:**
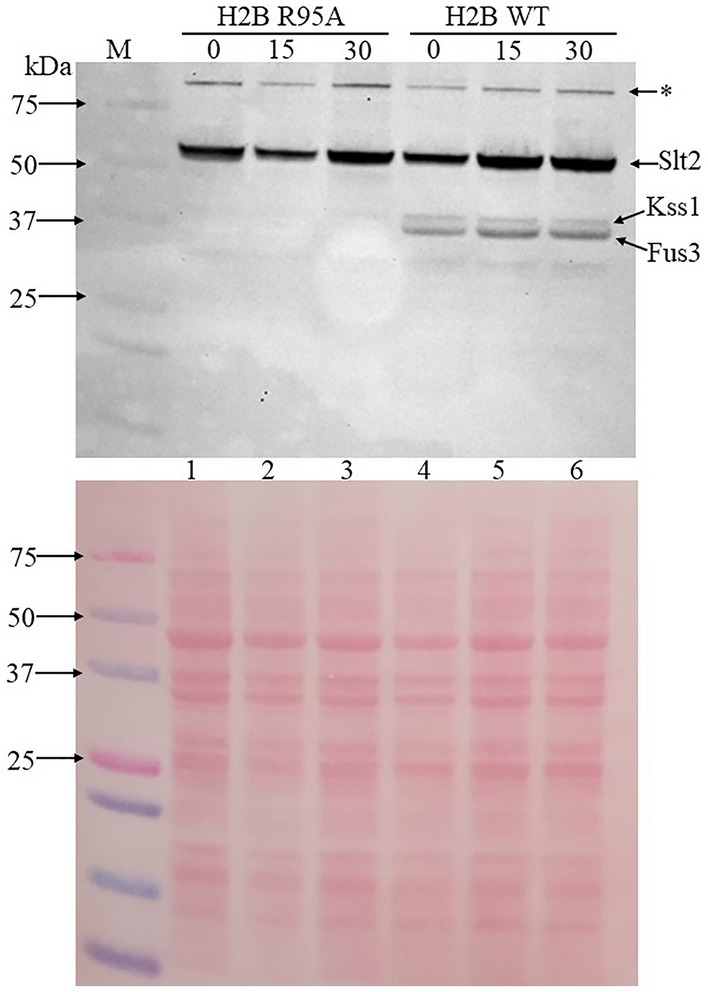
The MAP kinase Fus3, a downstream component of the pheromone pathway, is phosphorylated in the H2B WT, but not detected in the H2B R95A mutant. Exponentially growing cells were treated without (time zero) and with rapamycin (200 ng/ml). Samples were taken at 15 and 30 min for total protein extraction by trichloroacetic acid (TCA). The TCA extracted proteins were analyzed by immunoblot and probed with anti Erk1/2 to detect the MAPKs (see “Materials and methods”). The antibody detects three know proteins in yeast, the Slt2 kinase, Kss1, and Fus3. The asterisk denotes a high molecular weight polypeptide of unknown origin. The lower panel was stained with Ponceau to monitor for equal protein loading from the TCA samples. M, prestained protein markers in kDa.

The MAP kinases Kss1 (43 kDa) and Fus3 (41 kDa) are downstream components of the pheromone response pathway that can be phosphorylated^38^. Under conditions of starvation, Kss1 (43 kDa) can act on the Ste12 transcriptional activator to turn on filamentation genes^38^. Likewise, Fus3 when activated, for example, by α-factor can also regulate the function of the transcription factor Ste12 that controls expression of the mating genes leading to G_1_ arrest^38^. The phosphorylated form of Kss1 and Fus3 is readily detected by immunoblot analysis when probed with anti-ERK1/2 antibody that detects phosphorylated threonine and tyrosine^39^. This antibody recognizes the active phosphorylated form of Kss1 and Fus3, as well as other kinases such as Slt2 (52 kDa), which is required to maintain the integrity of the cell wall^39^. We examined the phosphorylation status of Kss1 and Fus3 and whether these two MAPKs would be altered in the H2B R95A mutant in comparison to the H2B WT in response to rapamycin. As expected, the anti-ERK1/2 antibody detected the phosphorylated Kss1 and Fus3 in the H2B WT strain using a 4–20% gradient SDS polyacrylamide gel, although better resolution can be seen on standard 12% gel (Fig. 6, lane 4 vs. lane 1) (for the full blot see Supplementary Fig. S7). Neither of the MAPKs was visibly detected in the H2B R95A mutant as compared to the H2B WT (Fig. 6, lane 1 vs. lane 4). This latter finding is consistent with the microarray data revealing that multiple genes of the pheromone pathway, including *FUS3*, are downregulated in the H2B R95A mutant (Fig. 2A). Treatment of the H2B WT cells with rapamycin did not show any noticeable increase in the Kss1 or Fus3 phosphorylation status (Fig. 6 lanes 5 and 6 vs. 4), excluding the possibility that rapamycin mode of action is similar to that of α-factor triggering MAPKs activation. It is noteworthy that the anti-ERK1/2 antibody can also detect the Slt2 kinase that responds to various stress conditions including heat shock and oxidative stress^39^. The Slt2 kinase was present at nearly the same level in the H2B WT and the H2B R95A mutant, and unaffected by rapamycin treatment (Fig. 6 lanes 4 to 6 vs. lanes 1 to 3), suggesting that the H2B R95A mutant is not likely to harbor global downregulation of signaling pathways. Taken together, the above results suggest that the inability of the H2B R95A mutant to arrest in the G_1_ phase in response to rapamycin is consistent with the downregulation of signaling components of the pheromone response pathway.

### Rapamycin treatment accumulates the cyclin Cln2 at a higher level in the *ste5Δ* mutant, as compared to the WT

**Figure 7:**
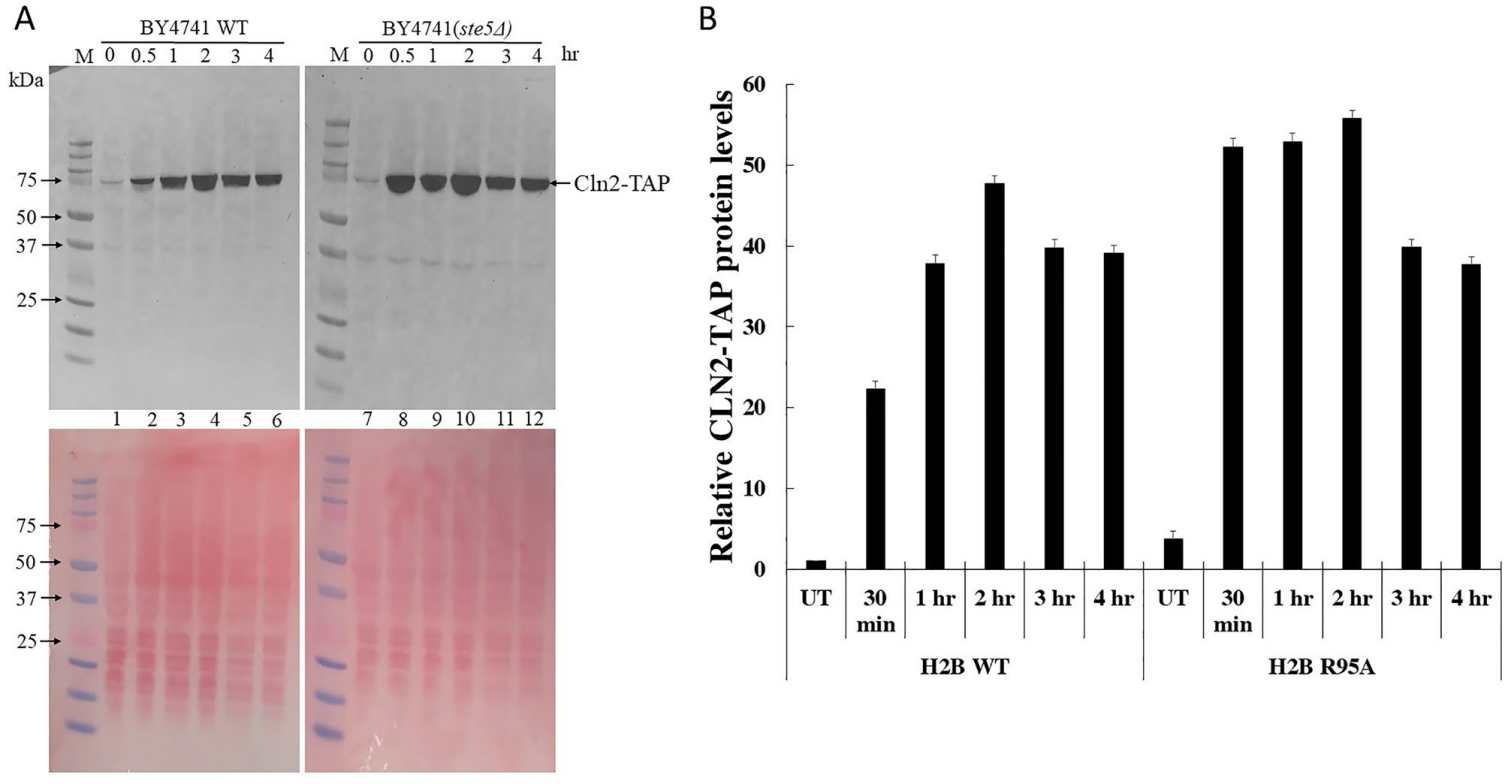
Rapamycin treatment accumulates cyclin Cln2 at a higher level in the *ste5Δ* mutant, as compared to the WT. (**A**) Exponentially growing cells derived from strain BY4741 (WT) carrying the TAP tag at the endogenous *CLN2* gene locus and the isogenic strain deleted for the *STE5* gene were treated without (time zero) and with rapamycin (200 ng/ml). Samples were taken at 30, 60, 120, 180 and 240 min for total protein extraction by TCA. The TCA extracted proteins were analyzed by immunoblot and probed with anti-TAP, which recognizes the protein A domain of the Cln2-TAP-tagged fusion protein (see “Materials and methods”). The lower panel was stained with Ponceau to monitor for equal protein loading from the TCA samples. M, prestained protein markers in kDa. (**B**) Quantification of Cln2-TAP levels relative to the untreated (zero time point). A repeat of this experiment was performed at time 0, 30 and 60 min and shown in Supplementary Fig. S10.

If rapamycin-induced G_1_ arrest involves the downregulation of the G_1_ cyclins (Cln1–3)^16,17^, we anticipate that such regulation would be compromised in the *ste5Δ* mutant allowing the cells to confer resistance to rapamycin. We examined the level of the G_1_ cyclin Cln2 in the parent BY4741 strain and the isogenic *ste5Δ* mutant, both expressing *CLN2-TAP* at the endogenous locus to monitor the level of Cln2-TAP with the TAP antibody, which detects the Protein A portion of the TAP tag^40^. Briefly, the parental and *ste5Δ* mutant cells were grown in liquid YPD and treated in the log phase without and with an acute dose of rapamycin (200 ng/ml), samples were withdrew at the following time points, zero, 30, 60, 120, 180, and 240 min, and processed by TCA extraction for immunoblot analysis. Upon rapamycin treatment, Cln2-TAP was unexpectedly and rapidly induced by more than 15-fold in the *ste5Δ* mutant within 30 min and maintain this induced level by 120 min, as compared to the time zero (untreated, UT) (Fig. 7A, lanes 7 to 10 and quantified as in Fig. 7B; see Supplementary Fig. S8 for the full immunoblot, Supplementary Fig. S9 for a lighter exposure, and Supplementary Fig. S10 for another independent experiment at zero, 30 and 60 min of rapamycin treatment). In contrast, there was a gradual induction of Cln2-TAP in the parental BY4741 strain after 30 min of rapamycin treatment, which continued to accumulate at 60 min and 120 min and by this time reached nearly comparable level as the *ste5Δ* mutant (Fig. 7A, lanes 1 to 4 vs. lanes 7 to 10; and quantified as in Fig. 7B; and see Supplementary Figs. S8, S9, and S10). These observations strongly indicate that the intense upregulation of Cln2 might be associated with the resistance of the *ste5Δ* mutant towards rapamycin. It is noteworthy that the peaked Cln2-TAP levels at 120 min in the parental BY4741 strain and the *ste5Δ* mutant began to slowly diminish at 180 min, consistent with previous findings that rapamycin treatment leads to the downregulation of G_1_ cyclins^16,17^ (see “Discussion”).

## Discussion

In the present study, we reported the identification and characterization of a variant of histone H2B, R95A, which was isolated by thoroughly screening a comprehensive histone mutant library^24^ for resistance specifically to the immunosuppressant rapamycin. The H2B R95A mutant exhibit marked resistance to rapamycin, as compared to other mutants identified by the screen that displayed varying resistance to the drug (Supplementary Fig. S3). We used this H2B R95A mutant to investigate the molecular defects associated with the Arg95Ala substitution leading to rapamycin resistance in yeast cells. Our data revealed that the H2B R95A variant downregulated many genes of the pheromone response pathway consistent with the mutant inability to arrest in the G_1_ phase in response to α-factor. Rapamycin is believed to trigger G_1_ arrest by repressing the transcription as well as the degradation of the G_1_ cyclins thereby blocking the G_1_ to S phase transition^10,17,18^. The observation that the H2B R95A mutant continued to proliferate in the presence of rapamycin strongly suggests that this phenotype is associated with the downregulation of components of the pheromone response pathway. In support of this notion, cells lacking the scaffold protein Ste5, a key component of the pheromone signaling pathway, replicate nearly the identical rapamycin-resistant phenotype as the H2B R95A mutant. As such, we conclude that the pheromone response pathway could be functionally involved in relaying the rapamycin signal for the subsequent G_1_ cell cycle arrest in WT cells. However, the exact link between the pheromone response pathway and the cell cycle arrest in response to rapamycin remains obscure. It is noteworthy that α-factor exposure triggers the sequential phosphorylation of the MAP kinases of the pheromone pathway including Fus3, which performs multiple functions such as repressing the expression of the cyclin genes to trigger G_1_ arrest^41^. We have not observed any visible activation of the MAPKs, Kss1 and Fus3 in the H2B WT strain upon rapamycin treatment using the anti-Erk1/2 antibody that recognizes an increase in the phosphorylation at the threonine and tyrosine residues of these kinases when cells are exposed to α-factor^38^. However, we cannot exclude the possibility that modification(s) other than phosphorylation of Kss1 and/or Fus3 might be involved in promoting rapamycin-induced G_1_ arrest.

It has been established that rapamycin treatment represses the transcription and translation of the G_1_ cyclins, which are normally required to target and promote degradation of inhibitors such as Whi5 and Sic1 of transcription factors ultimately setting the transcription factors free to activate gene expression^10,16,42^. Several studies demonstrated that rapamycin triggers the downregulation of G_1_ cyclins leading to G_1_ arrest^16,17^. While the downregulation of the G_1_ cyclins can be detected as early as 60 min and near-complete disappearance by 240 min, which appears to be strain-dependent^17,18^, paradoxically, we found a different response in the commonly used parental background strain BY4741. Cln2 monitored as Cln2-TAP, was significantly and gradually induced by rapamycin from 30 min and lasted for 120 min in the BY4741 WT strain followed by a slow degradation detectable at ~ 180 min (Fig. 7). Nonetheless, under the same growth condition cells lacking the scaffold protein Ste5 showed a rapid and substantially elevated level of Cln2 as early as 30 min following treatment with rapamycin when compared to the gradual induction seen in the WT strain. For both strains, the disappearance of Cln2-TAP was noticeable at 180 min, although we and others have not conducted pulse-chase experiments whereby cells are treated with rapamycin for 30 min, wash free of the drug, and then monitor the rate of disappearance of Cln2-TAP (Fig. 7).

**Figure 8:**
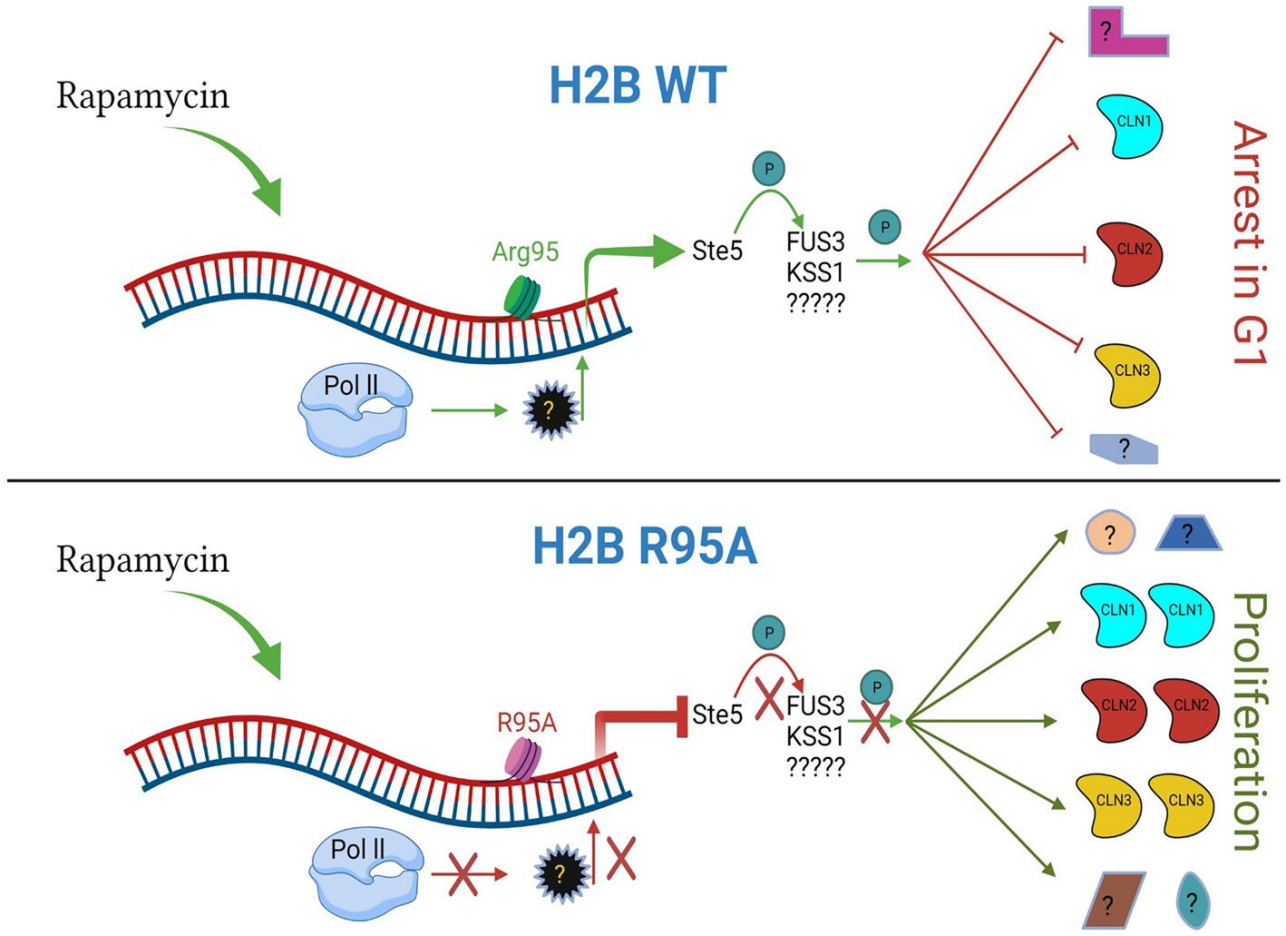
A model illustrating the importance of H2B R95 residue in maintaining the expression of the pheromone genes required to arrest cells in the G_1_ phase in response to rapamycin. The native H2B R95 residue is required to recruit RNA polymerase II and the associated transcriptional activator under normal conditions to maintain the expression of the pheromone response genes. Recruitment of RNA polymerase II and the associated transcriptional activator is defective by the H2B R95A mutation preventing the expression of the pheromone response genes. This allows the dysregulation of the G_1_ cyclins allowing expression of Cln1, Cln2, and perhaps Cln3, as well as other factors to promote cell proliferation in the presence of rapamycin. The illustration was created with features from BioRender (BioRender.com).

The rapid induction and the accumulation of higher Cln2 level upon rapamycin treatment could be due to the loss of negative feedback control in the absence of Ste5^43^. Consequently, this elevated Cln2 level could reinforce phosphorylation of the CDK inhibitor Sic1 such that it is rapidly degraded and unblock any potential cell cycle arrest caused by rapamycin^43^. This might be a likely scenario as Ste5 and Sic1 possess the Leucine-Proline rich sequence required to allow docking of Cln2 to regulate the functionality of these substrates^44^. Another possibility might be to maintain the transcriptional repressor Whi5 in the hyperphosphorylated state preventing it from inhibiting the SBF transcription factor that activates *CLN1* and *CLN2* gene expression, thus creating a positive feedback loop that drives higher expression of Cln1, as well as Cln2 and perhaps other factors crucial for cell cycle progression^15^. In the case of the parent, we cannot exclude the possibility that the gradual increase in Cln2 level caused by rapamycin might perform a significant function such as regulating Ste5 membrane localization by Cln2-CDK^37,45^. Nevertheless, the most significant finding from our study is the discovery that yeast cells exploit the pheromone pathway to arrest the cell cycle in the G_1_ phase in response to rapamycin (Fig. 8). We predict that deleting the MAPK genes, *KSS1* and *FUS3*, would promote the expression of the G_1_ cyclins including Cln1, Cln2, and possibly Cln3, and allow the cells to proliferate in the presence of rapamycin, although we cannot exclude the involvement of other factors (Fig. 8). We have tried to modulate this pathway by driving the overexpression of Ste5, as a Ste5-MYC tag expressed from the independent constitutive promoter *ADH*, and found no significant effect on the response of either the H2B WT or the H2B R95A mutant towards rapamycin when compared to the empty vector (see Supplementary Fig. S11A for Ste5-MYC expression; S11B for the full blot; and S11C for the sensitivity to rapamycin by spot test analysis for three independent experiments). So far, we have no clear explanation why the overexpression of Ste5-MYC did not alter the rapamycin response in the strains unless other factors are becoming limiting and thus unable to activate Ste5-MYC and or to execute Ste5-MYC signal, as in the case of the H2B R95A mutant lacking proper expression of 25 other components including *STE18*, *STE6*, *FAR1*, and the MAPKs *KSS1* and *FUS3*, of the pheromone pathway.

The observation that the *ste5Δ* mutant, but not the *ste2Δ* strain lacking the α-factor receptor, displayed resistance to rapamycin, suggests that the mechanism by which rapamycin transmits a signal via the pheromone pathway precludes the recruitment of Ste5 to the Ste2 receptor on the plasma membrane as reported for α-factor. Thus, while α-factor binds to Ste2 and initiates the signal to arrest the cell cycle in G_1_, rapamycin could intersect the pathway at the level of the Ste5 scaffold protein or further downstream. It is noteworthy that Ste5 is phosphorylated at eight sites within the N-terminus and these can be regulated by CDK and the MAP kinase Fus3 to modulate Ste5 membrane association^34,45^. We have not seen changes in the migration pattern of Ste5-GFP in response to rapamycin, although the addition or removal of any unique phosphorylation might be technically challenging to be discerned by one-dimensional SDS-PAGE. There is evidence that during the mating signal Ste5 undergoes conformational changes to unblock its auto-inhibitory effect caused by an intramolecular interaction between two domains, the pleckstrin homology, and the von Willebrand type A, thereby allowing the activation of Fus3^46^. It would be interesting to explore whether the inhibition of the Torc1 kinase by rapamycin intersects the pheromone response pathway to alter the phosphorylation sites within the N-terminus of Ste5 and or unblock its auto-inhibitory conformation such that it is poised to activate G_1_ arrest.

We remark that the H2B R95A variant was also previously isolated in a screen for the loss of transcription repression of reporter genes embedded within the silent chromatin regions of the telomere^32^. Dai et al. showed that the H2B R95A mutant lost telomeric silencing in a region that spans at least 20-kb from the end of the telomere^32^. In addition, the authors described other histone residues such as H2B K123, which is ubiquitylated and required for the di- and trimethylation of H3 K4 and H3 K79 that caused a loss in silencing at rDNA and telomeres when mutated^32^. We did not isolate from our screen the H2B K123A, H3 K4A, H3 K79A, or other known histone mutants, such as H4 K16 that lost silencing at the rDNA and telomeres, that would play a role in the resistance to rapamycin^32^. Thus, while defective silencing at the ends of the telomere may lead to other unidentified drug-resistant phenotypes, the telomeric silencing caused by H2B R95A is not associated with rapamycin resistance, otherwise, several of the histone variants causing silencing at rDNA and the telomeres would be expected to show resistance to rapamycin. We propose that the Arg95 residue of histone H2B performs multiple functions including the ability to maintain expression of the pheromone pathway genes, which are required to mount a response to rapamycin-induced cell cycle arrest.

Attempts to determine whether the Arg95 residue is modified using mass spectrometry analysis of purified H2B derived from untreated and rapamycin-treated cells did not reveal any modification. Dai et al. also searched for possible Arg95 methylation and found no evidence for such modification under normal growth conditions^32^. We cannot exclude the possibility that a minor fraction of the total H2B could be methylated at Arg95, in particular since H2B R95A does not have a global effect and only affected the expression of a small number of genes. There are several examples of a unique histone modification that alters a specific set of genes, which affects a unique biological process. For example, the study by Tessarz et al. used a biochemical approach and identified a single glutamine residue (Q105) of yeast H2A that is methylated by the essential Nop1 methyltransferase^47^. The H2A Q105 methylation is restricted to the nucleolus and located primarily at the 35S DNA transcription units, as compared to the entire yeast genome^47^. H2A Q105 spans a binding site for the FACT complex and H2A Q105A mutant can disrupt this binding altering transcription at the rDNA locus^47^. So far, the transcription factor(s) that is recruited by H2B Arg95 to promote expression of the pheromone response gene is not known.

Besides H2B R95A, our screen also revealed mutants that are less resistant to rapamycin such as H4 L22A. Although the N-terminal of H4 is linked to rDNA regulation, silencing of this region is not involved in rapamycin resistance. We have not explored if H4 L22A plays a minor role in activating the pheromone pathway or performs an independent function leading to rapamycin resistance. However, several other proteins are involved in rapamycin resistance such as the copper-zinc superoxide dismutase Sod1^48^. In the absence of Sod1, it is believed that the Torc1 complex is inactivated by the elevated levels of endogenous superoxide anions leading to rapamycin resistance^48^. Since *sod1Δ* mutants cause oxidative damage to other metabolic enzymes^49^, multiple pathways may likely co-exist besides the Torc1 complex and the downregulation of the pheromone pathway to cause rapamycin resistance.

## Supplementary Information

Supplementary Information.

## Data Availability

The datasets analyzed in this study are available in the GEO DataSets repository, with the accession number GSE201413 (https://www.ncbi.nlm.nih.gov/geo/query/acc.cgi?acc=GSE201413) and available publicly on April 25, 2022.
