# Supplementary material for: The histone H2B Arg95 residue links the pheromone response pathway to rapamycin-induced G1 arrest in yeast
Source: Sci Rep. 2022 Jun 15;12:10023. doi: 10.1038/s41598-022-14053-9 (PMC9200821; doi:10.1038/s41598-022-14053-9)
Supplement: Supplementary file 1 — Supplementary Information. [file 41598_2022_14053_MOESM1_ESM.docx]

**The Histone H2B Arg95 residue links the Pheromone Response Pathway to Rapamycin-Induced G_1_ Arrest in Yeast**

Abdallah Alhaj Sulaiman^1^, Reem Ali^1^, Mustapha Aouida^1^, Balasubramanian Moovarkumudalvan^1^, and Dindial Ramotar^1, a^

^1^Division of Biological and Biomedical Sciences, College of Health and Life Sciences, Hamad Bin Khalifa University, Education City, Qatar Foundation, Doha, Qatar, P.O.Box: 34110

^a^ Corresponding author Email: dramotar@hbku.edu.qa

**Table S1.**  Yeast strains and mutants used in this study

| **Strains** | **Genotype** | **Source** |
| --- | --- | --- |
| BY4741 | Parent, *Mat a, his3-1, leu2-0, met15-0, ura3-0* | Euroscarf (Frankfurt, Germany) |
| *ste5Δ::KANMX* | isogenic to BY4741, except deleted for the *STE5* | This work (resistant to rapamycin) |
| *ste2Δ::KANMX* | isogenic to BY4741, except deleted for the *STE2* | This work |
| FY406 | The H2A/H2B collection is in FY406 MATa (hta1-htb1)Δ::LEU2, (hta2-htb2) Δ::TRP1, his3 Δ 200 leu2 Δ 1 ura3-52 trp1 Δ 63 lys2-128 Δ <pSAB6 (HTA1-HTB1-URA3). The covering plasmid for H2A/H2B uses the His marker. | Ali Shilatifard (Kansas, USA) |
| YBL574 | The H3/H4 collection is in Winston’s YBL574 (MATa, leu2Δ1, his3Δ200, ura3-52, trp1Δ63, lys2-128δ, (hht1-hhf1)ΔLEU2 (hht2-hhf2)Δ::HIS3 Ty912Δ35-lacZ::his4, {pDM9-HHT1-HHF1-URA3}. The covering plasmid for H3/H4 uses the Trp marker. | Ali Shilatifard |
| *TAP* tag strains collection | MATa his3Δ1 leu2Δ0 met15Δ0 ura3Δ0 | Dharmacon |
| Y1a | Parent W303 (leu2-3, 112, trp1-1, can1-100, ura3-1, ade2-1, his3-11,15) | Lab strain |
| *ste5Δ::LEU2* | Isogenic to W303, except deleted for *STE5* | This work (resistant to rapamycin) |

**Table S2. GO analysis for genes differentially regulated in the H2B WT and H2B R95A in untreated and rapamycin treated**

| Gene Ontology (GO) terms significantly represented among up-regulated genes in H2B wild type in rapamycin treatment | | | | | |
| --- | --- | --- | --- | --- | --- |
| Biological Pathways | GO term | Genes | Fold Enrichment | p Value | FDR |
| Organic acid transport | GO:0015849 | DAL5, GAP1, DIP5, CAN1, AGP1, PTR2 | 19.55823 | 6.71E-06 | 0.007968 |
| Amine transport | GO:0015837 | GAP1, DIP5, DUR3, CAN1, AGP1 | 23.32375 | 3.84E-05 | 0.045602 |
| Carboxylic acid transport | GO:0046942 | DAL5, GAP1, DIP5, CAN1, AGP1 | 19.3254 | 8.09E-05 | 0.096019 |
| Amino acid transport | GO:0006865 | GAP1, DIP5, CAN1, AGP1 | 21.64444 | 6.26E-04 | 0.740503 |
|  |  |  |  |  |  |
| Gene Ontology (GO) terms significantly represented among down-regulated genes in H2B wild type under rapamycin treatment | | | | | |
| Biological Pathways | GO term | Genes | Fold Enrichment | p Value | FDR |
| rRNA processing | GO:0006364 | RRB1, GAR1, UTP15, CSL4, UTP14, UTP13, SAS10, ESF2, MAK16, IMP4, RRP3, RPS9A, RRP1, EMG1, MPP6, RRP8, RRP9, NOC2, BUD22, RCL1, BUD23, MRT4, ENP1, NOP8, BFR2, NHP2, UTP8, SNU13, NSR1, FCF2, SOF1, EBP2, DRS1, DIP2, KRI1, SLX9, RRP14, RRP17, RLP7, RRP15, NOP53, HCA4, RPF2, PWP1, NOP15, NOP12, NOP16, DBP3, DBP6, FYV7 | 13.23154 | 4.38E-47 | 5.87E-44 |
| Ribosome biogenesis | GO:0042254 | SSF1, RRB1, GAR1, UTP15, CSL4, UTP14, UTP13, SAS10, ESF2, MAK16, IMP4, RRP3, RPS9A, RRP1, EMG1, MPP6, RRP8, RRP9, NOC2, BUD22, RCL1, BUD23, MRT4, ENP1, NOP8, BFR2, CIC1, REI1, LTV1, NHP2, UTP8, SNU13, NSR1, FCF2, EBP2, SOF1, DRS1, DIP2, KRI1, SLX9, RRP14, RRP17, RLP7, RRP15, NOP53, HCA4, RPF2, PWP1, NOP15, NOP12, NOP16, DBP3, RLP24, DBP6, FYV7 | 9.91046 | 6.79E-46 | 9.10E-43 |
| RNA processing | GO:0006396 | RRB1, GAR1, UTP15, CSL4, UTP14, UTP13, SAS10, ESF2, MAK16, IMP4, RRP3, RPS9A, RRP1, EMG1, MPP6, RRP8, RRP9, NOC2, BUD22, RCL1, BUD23, MRT4, TRM82, ENP1, NOP8, BFR2, CIC1, NHP2, TRM1, UTP8, SNU13, NSR1, FCF2, TRM8, EBP2, SOF1, DRS1, DIP2, KRI1, SLX9, RRP14, TRM10, RRP17, RLP7, RRP15, NOP53, HCA4, RPF2, PWP1, NOP15, NOP12, NOP16, DUS3, DBP3, DBP6, FYV7 | 6.877317 | 5.19E-38 | 6.95E-35 |
| Maturation of SSU-rRNA from tricistronic rRNA transcript (SSU-rRNA, 5.8S rRNA, LSU-rRNA) | GO:0000462 | RRP3, RPS9A, EMG1, UTP8, UTP15, SNU13, UTP14, FCF2, UTP13, SAS10, RCL1, SOF1, ESF2, BUD23, ENP1, DIP2, KRI1, SLX9, FYV7 | 14.47817 | 1.19E-16 | 1.44E-13 |
| Ribosome assembly | GO:0042255 | SSF1, DRS1, ESF2, RLP7, DBP3, NSR1, RLP24, DBP6, RPF2, NOC2 | 9.166196 | 9.05E-07 | 0.001213 |
| Ribosome export from nucleus | GO:0000054 | BUD23, NOP53, LTV1, REI1, NOC2 | 7.354274 | 0.004264 | 5.566975 |
| tRNA methylation | GO:0030488 | TRM10, TRM1, TRM82, TRM8 | 14.05483 | 0.002518 | 3.323055 |
| Cytokinesis during cell cycle | GO:0033205 | BUD22, NOP15, ATC1, BUD23, BUD27 | 4.273429 | 0.027694 | 31.36973 |
|  |  |  |  |  |  |
| Gene Ontology (GO) terms significantly represented among up-regulated genes in R95A mutant under rapamycin treatment | | | | | |
| Biological Pathways | GO term | Genes | Fold Enrichment | p Value | FDR |
| Ammonium transport | GO:0015696 | MEP2, MEP1, ATO2 | 43.48214 | 0.001825 | 2.417298 |
| Organic cation transport | GO:0015695 | MEP2, MEP1, ATO2 | 32.61161 | 0.003357 | 4.405357 |
| Organic acid transport | GO:0015849 | PXA1, DAL5, GAP1, DIP5, CAN1, PTR2 | 6.286575 | 0.002272 | 3.000955 |
| Amine transport | GO:0015837 | GAP1, DIP5, DUR3, CAN1 | 5.997537 | 0.027162 | 30.85116 |
| Cofactor catabolic process | GO:0051187 | HMX1, CIT2, CIT3, IDP3 | 9.938776 | 0.00691 | 8.871038 |
| Response to temperature stimulus | GO:0009266 | SPL2, SRL3, IKS1, CRG1, AFR1, TKL2, HBT1 | 2.8053 | 0.03428 | 37.33028 |
|  |  |  |  |  |  |
| Gene Ontology (GO) terms significantly represented among down-regulated genes in R95A mutant under rapamycin treatment | | | | | |
| Biological Pathways | GO term | Genes | Fold Enrichment | p Value | FDR |
| Ribosome biogenesis | GO:0042254 | RPS8A, RPS8B, SSF1, ARX1, EFG1, RRB1, MRD1, NOG1, NOG2, NUG1, ESF1, RPS27B, IMP3, ESF2, MAK16, ALB1, PXR1, ERB1, CBF5, IMP4, RPS27A, RPS7B, MAK11, RPS9A, RRP3, KRR1, RPS9B, FAL1, RRP1, EMG1, RRP5, RPS0B, SPB1, RPS0A, RRP8, RRP9, SPB4, KRE33, RSA4, RCL1, RPL8A, NOP1, RPS26B, NOP2, NOP4, HAS1, MRT4, RSA3, NOP6, MPP10, NOP7, NOP8, RPS13, NOP9, CIC1, MAK21, RPL8B, ECM16, NAN1, RPS11A, RPS11B, YAR1, BRX1, PUS7, RPS1B, EBP2, SOF1, DRS1, DIP2, RPL5, BCP1, KRI1, SLX9, RPS19A, RPS19B, UTP30, UTP23, UTP21, RPS10B, TSR1, TSR2, RPS10A, RNT1, ECM1, RPF2, NOP10, RPF1, NOP14, ROK1, TMA23, NOP15, NOP12, NOP16, DBP2, RPL6A, DBP3, RRS1, RPL6B, DBP6, RPS18A, RPS18B, FYV7, DBP9, MTR3, RPS17B, MTR4, YTM1, UTP18, GAR1, UTP15, UTP14, RPS2, UTP11, CMS1, UTP10, UTP13, DBP10, RPS3, RPL12A, SAS10, RPL12B, RLI1, URB2, URB1, RPS23A, RPS23B, RPS16A, NIP7, FAP7, MPP6, DIM1, NOC4, NOC2, RPS16B, NOC3, BUD22, BUD21, RPL11A, BUD23, RPL11B, ENP1, ENP2, LCP5, PRP43, BFR2, TIF5, REI1, LTV1, NHP2, RPS21A, UTP4, UTP7, UTP8, UTP5, UTP6, NOB1, SNU13, UTP9, NSR1, SDA1, FCF2, BMS1, TRM7, RPL30, LSG1, NSA2, NMD3, RPL40B, RRP14, MAK5, RIX7, RRP12, IPI1, RRP17, RIX1, RLP7, PNO1, RPP1, RPS14B, HCA4, CGR1, RPL40A, PWP2, RPS7A, PWP1, RPS6A, RPS6B, NOP58, YVH1, RPS24A, IPI3, RLP24, RPS24B | 8.028096 | 1.57E-143 | 2.37E-140 |
| rRNA metabolic process | GO:0016072 | RPS8A, RPS8B, EFG1, RRB1, MRD1, NOG1, NUG1, ESF1, RPS27B, IMP3, ESF2, MAK16, PXR1, ERB1, CBF5, IMP4, RPS27A, RPS7B, MAK11, RPS9A, RRP3, KRR1, FAL1, RPS9B, RRP1, EMG1, RRP5, RPS0B, SPB1, RPS0A, RRP8, RRP9, SPB4, RCL1, NOP1, NOP2, NOP4, HAS1, MRT4, NOP6, MPP10, NOP7, NOP8, RPS13, NOP9, ECM16, NAN1, RPS11A, RPS11B, BRX1, PUS7, RPS1B, EBP2, SOF1, DRS1, DIP2, KRI1, SLX9, UTP30, UTP23, UTP21, TSR1, TSR2, AIR1, RNT1, RPF2, NOP10, RPF1, NOP14, ROK1, TMA23, NOP15, NOP12, NOP16, DBP2, RRS1, DBP3, DBP6, RPS18A, RPS18B, FYV7, DBP9, MTR3, MTR4, YTM1, UTP18, GAR1, UTP15, UTP14, RPS2, UTP11, UTP10, UTP13, DBP10, SAS10, RLI1, URB2, URB1, RPS23A, RPS23B, RPS16A, NIP7, FAP7, MPP6, DIM1, NOC4, NOC2, RPS16B, NOC3, BUD22, BUD21, BUD23, ENP1, ENP2, LCP5, PRP43, BFR2, NHP2, RPS21A, UTP4, UTP7, UTP8, UTP5, UTP6, NOB1, SNU13, UTP9, NSR1, FCF2, BMS1, TRM7, RPL30, NSA2, RRP14, MAK5, RRP12, IPI1, RRP17, RIX1, RLP7, PNO1, RPP1, RPS14B, HCA4, CGR1, PWP2, RPS7A, PWP1, RPS6A, RPS6B, NOP58, RPS24A, IPI3, RPS24B | 9.077526 | 2.02E-120 | 3.04E-117 |
| RNA processing | GO:0006396 | RPS8A, RPS8B, EFG1, RRB1, MRD1, NOG1, NUG1, ESF1, RPS27B, IMP3, ESF2, MAK16, PXR1, ERB1, CBF5, IMP4, RPS27A, RPS7B, MAK11, RPS9A, RRP3, KRR1, RPS9B, FAL1, RRP1, EMG1, RRP5, RPS0B, SPB1, RPS0A, RRP8, RRP9, SPB4, RCL1, NOP1, NOP2, NOP4, HAS1, MRT4, NOP6, MPP10, NOP7, NOP8, RPS13, NOP9, CIC1, ECM16, NAN1, RPS11A, PUS1, RPS11B, BRX1, PUS7, LHP1, RPS1B, EBP2, SOF1, DRS1, DIP2, KRI1, SLX9, UTP30, UTP23, UTP21, TSR1, TSR2, AIR1, RNT1, RPF2, NOP10, RPF1, NOP14, ROK1, TMA23, NOP15, NOP12, NOP16, DBP2, DBP3, RRS1, DBP6, RPS18A, RPS18B, FYV7, DBP9, MTR3, MTR4, YTM1, UTP18, GAR1, UTP15, UTP14, RPS2, UTP11, UTP10, UTP13, NCS2, DBP10, SAS10, RLI1, URB2, URB1, RPS23A, RPS23B, RPS16A, ELP3, ELP2, ELP6, NIP7, FAP7, MPP6, DIM1, NOC4, NOC2, RPS16B, NOC3, BUD22, BUD21, BUD23, GCD10, ENP1, TRM82, ENP2, LCP5, SUA5, PRP43, BFR2, NHP2, RPS21A, UTP4, TRM3, TRM1, UTP7, UTP8, TRM2, UTP5, UTP6, NOB1, SNU13, UTP9, NSR1, TRM9, FCF2, BMS1, TRM7, TRM8, NCL1, RPL30, NSA2, RRP14, MAK5, TRM11, TRM10, RRP12, IPI1, RRP17, RIX1, RLP7, PNO1, RPP1, RPS14B, HCA4, CGR1, PWP2, RPS7A, PWP1, RPS6A, RPS6B, DUS3, NOP58, RPS24A, IPI3, RPS24B | 4.936313 | 1.36E-84 | 2.04E-81 |
| Maturation of SSU-rRNA | GO:0030490 | RPS8A, RPS8B, EFG1, UTP18, MRD1, UTP15, UTP14, UTP11, UTP10, UTP13, SAS10, RPS27B, ESF2, RPS23A, RPS23B, RPS27A, RPS16A, KRR1, RPS9A, RRP3, RPS9B, FAL1, EMG1, RRP5, RPS0B, RPS0A, FAP7, NOC4, RPS16B, RCL1, BUD21, NOP1, BUD23, MPP10, NOP7, ENP1, RPS13, NOP9, PRP43, ECM16, RPS21A, NAN1, UTP4, UTP7, RPS11A, UTP8, RPS11B, UTP5, UTP6, NOB1, SNU13, UTP9, FCF2, RPS1B, SOF1, DIP2, KRI1, SLX9, UTP23, RRP12, TSR2, PNO1, RPS14B, PWP2, NOP14, RPS6A, RPS6B, RRS1, NOP58, RPS24A, RPS24B, RPS18A, RPS18B, FYV7 | 12.0714 | 8.04E-64 | 1.21E-60 |
| Ribosome assembly | GO:0042255 | SSF1, RPS17B, RPS11A, RPS11B, MRD1, NSR1, BRX1, NOG1, SDA1, BMS1, DBP10, RPL12A, RPS27B, RPL12B, DRS1, ESF2, URB1, RPL5, RPS27A, MAK11, NMD3, MAK5, IPI1, RIX1, NIP7, RLP7, RPS0B, RPS14B, RPS0A, SPB4, RPF2, RPF1, NOC2, RSA4, RPL11A, RPL11B, HAS1, RSA3, RPL6A, DBP3, YVH1, RPL6B, IPI3, MAK21, DBP6, TIF5, RLP24, DBP9 | 10.20964 | 1.07E-37 | 1.61E-34 |
| Regulation of translation | GO:0006417 | RPS8A, RPG1, RPS8B, CAF20, RPS2, SXM1, RPL12A, RPL12B, RLI1, PUF6, NIP1, RPS23A, RPS23B, RPS16A, RPS9A, RPS9B, RPL4B, RPS0B, RPS0A, CDC60, RPL31B, PRT1, RPS16B, RPL16A, RPL8A, GCD10, PRO1, TIF5, RPL8B, RPS11A, RPS11B, RPL20B, RPL20A, RPL27B, WRS1, RPL24A, ARO4, RPS1B, SRO9, RPL30, RPL17B, RPL14B, RPL5, RBG1, RPL2B, URA7, RPL2A, RPS14B, PWP1, MES1, RPL18A, RPL18B, RPL13A, NEW1, RPL6A, DBP2, RPL6B, RPS24A, RPS24B | 4.254018 | 3.62E-19 | 5.44E-16 |
| Posttranscriptional regulation of gene expression | GO:0010608 | RPS8A, RPG1, RPS8B, CAF20, RPS2, SXM1, RPL12A, RPL12B, RLI1, PUF6, NIP1, RPS23A, RPS23B, RPS16A, RPS9A, RPS9B, RPL4B, RPS0B, RPS0A, CDC60, RPL31B, PRT1, RPS16B, RPL16A, RPL8A, GCD10, PRO1, TIF5, RPL8B, RPS11A, RPS11B, RPL20B, RPL20A, RPL27B, WRS1, RPL24A, ARO4, RPS1B, SRO9, RPL30, RPL17B, RPL14B, RPL5, RBG1, RPL2B, URA7, RPL2A, RPS14B, PWP1, MES1, RPL18A, RPL18B, RPL13A, NEW1, RPL6A, DBP2, RPL6B, RPS24A, RPS24B | 3.988142 | 6.94E-18 | 1.04E-14 |
| Regulation of cellular protein metabolic process | GO:0032268 | RPS8A, RPG1, RPS8B, CAF20, RPS2, SXM1, RPL12A, RPL12B, RLI1, PUF6, NIP1, RPS23A, RPS23B, RPS16A, RPS9A, RPS9B, RPL4B, RPS0B, RPS0A, CDC60, RPL31B, PRT1, RPS16B, RPL16A, RPL8A, GCD10, PRO1, TIF5, RPL8B, RPS11A, RPS11B, RPL20B, RPL20A, RPL27B, WRS1, RPL24A, FPR4, ARO4, RPS1B, SRO9, RPL30, RPL17B, RPL14B, RPL5, RBG1, RPL2B, URA7, RPL2A, RPS14B, PWP1, MES1, RPL18A, RPL18B, RPL13A, NEW1, RPL6A, DBP2, RPL6B, RPS24A, RPS24B | 3.88576 | 9.90E-18 | 1.49E-14 |
| tRNA methylation | GO:0030488 | NCL1, TRM3, TRM11, TRM10, TRM1, GCD10, TRM82, TRM9, TRM7, TRM8 | 8.508036 | 5.41E-07 | 8.13E-04 |
| Ribosome export from nucleus | GO:0000054 | IPI1, RIX7, RIX1, FAP7, NOG1, SDA1, NOG2, ECM1, RPF1, NOC2, RSA4, RLI1, BUD23, LSG1, RRS1, NOP9, BCP1, REI1, LTV1, NMD3 | 7.123007 | 3.23E-12 | 4.86E-09 |

**
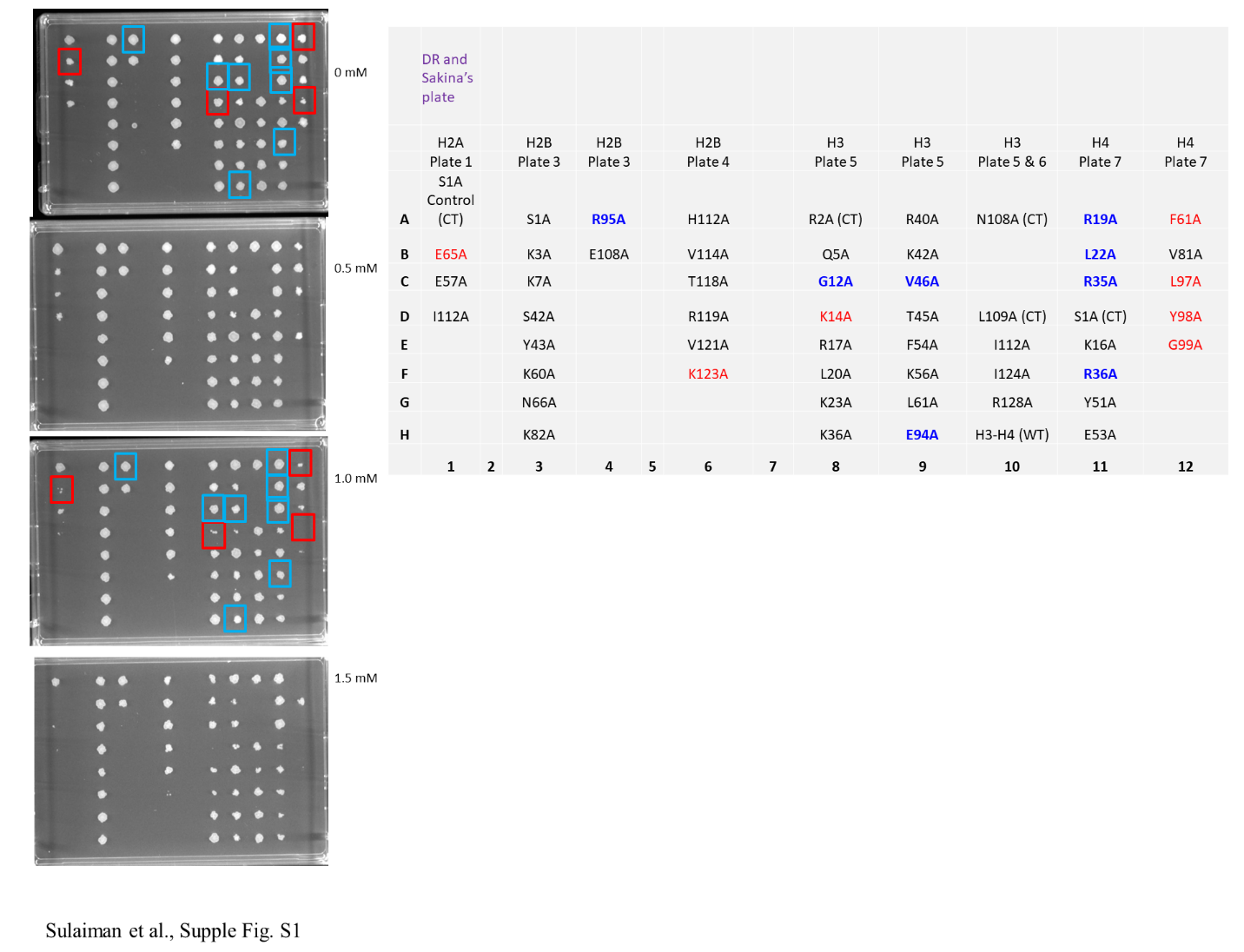
**

**Figure S1**: **Responses of the indicated histone mutants towards arsenite**. Cultures adjusted to OD 600 nm of 1.0 were spotted onto solid YPD plates without and with increasing concentrations of arsenite. Plates were photographed after 48 hours of incubation at 30 ^o^C. The arsenite-sensitive mutants are enclosed in red squares. The rapamycin-resistant mutants that were tested against arsenite are shown in blue squares.

**
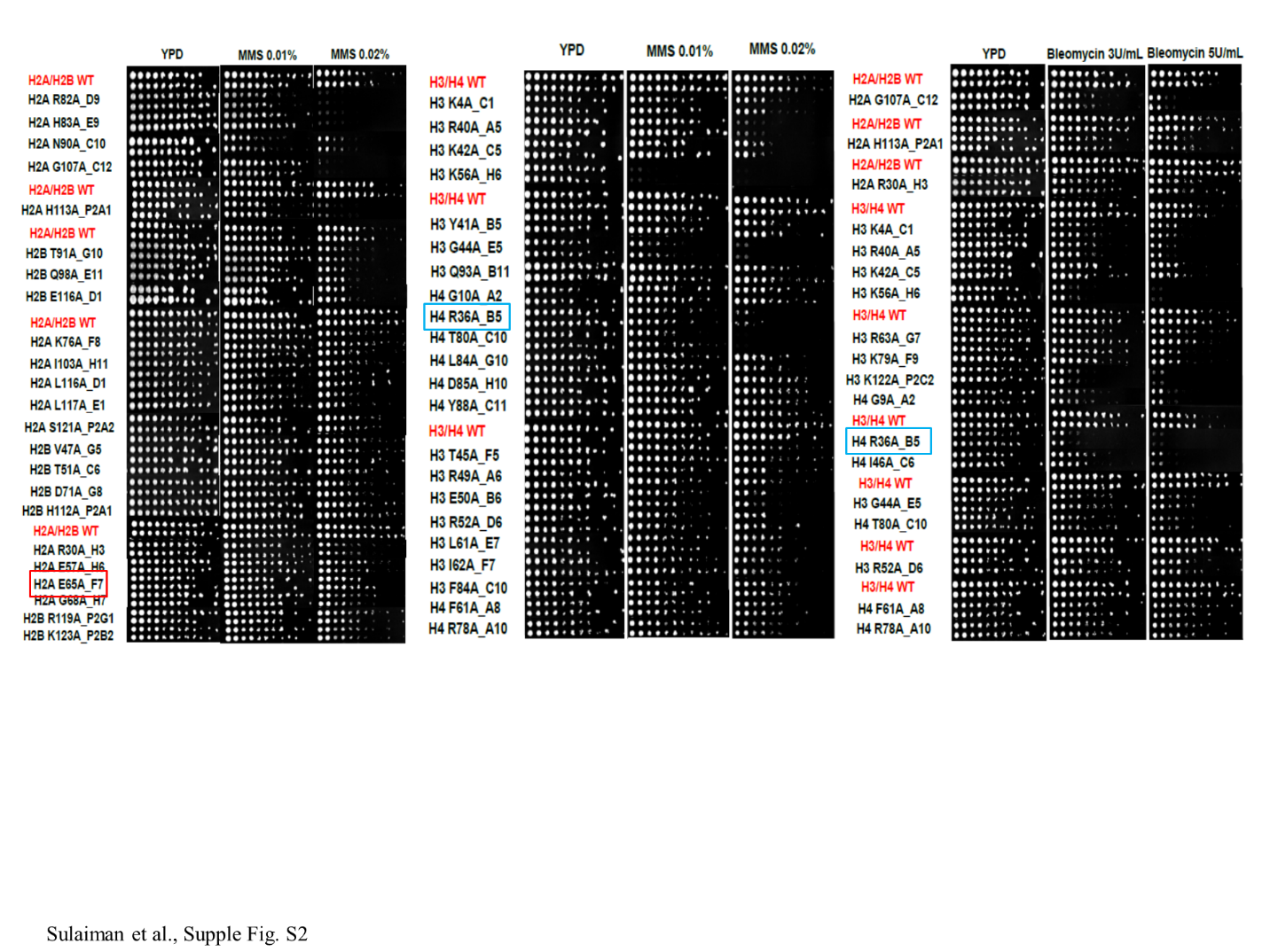
**

**Figure S2**: **The histone mutants displaying sensitivity to methyl methanesulfonate (MMS) and bleomycin (BLM)**. The entire histone mutant collection was examined by 4-spot assay for those variants exhibiting sensitivity to the DNA damaging agents MMS and or BLM (Aouida et al 2004). The data were generously provided by Dr. Shima Nakanishi from Dr. Ali Shilatifard laboratory (Kansas City, Missouri, USA). The histone H2A mutant H2A E65A highlighted in the red rectangle and exhibiting sensitivity to rapamycin, also displayed sensitivity to MMS. The H4 mutant H4 R36A highlighted in the blue rectangle and exhibiting resistance to rapamycin (see supplementary Figure. S3 below) displayed sensitivity to both MMS and BLM.

**
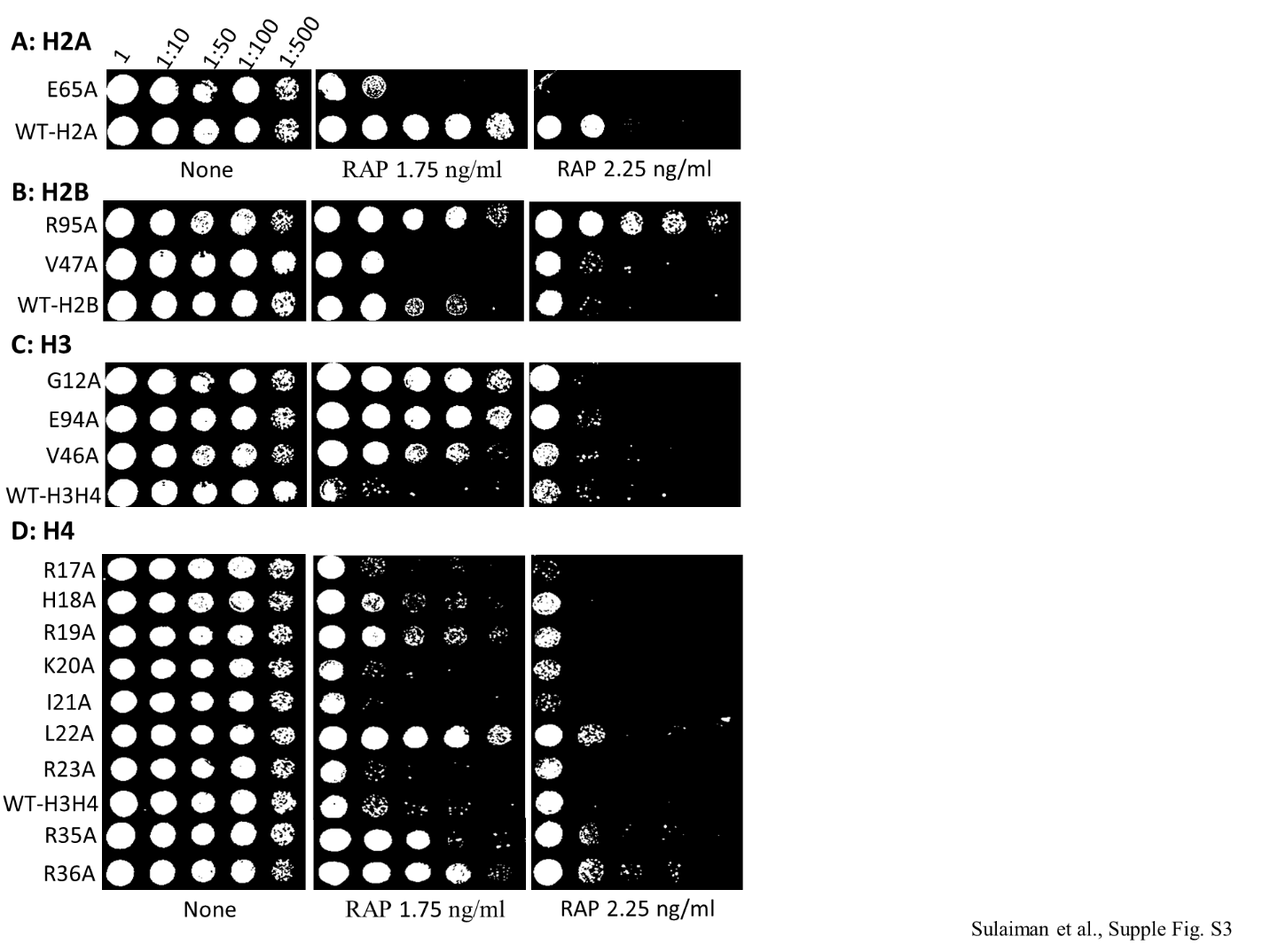
**

**Figure S3**: **Responses of the indicated histone mutants towards rapamycin**. Cultures adjusted to OD 600 nm of 1.0 were serially diluted and spotted onto solid YPD plates without and with increasing concentrations of rapamycin. Plates were photographed after 48 hours of incubation at 30 ^o^C.

NB: the mutant H4 L22A is less resistant to rapamycin than H2B R95A in three independent experiments.


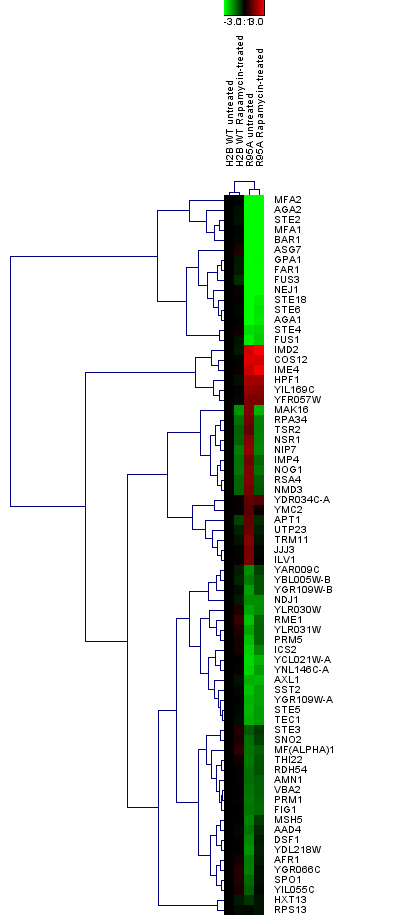


**Figure S4: Heat map visualization obtained by hierarchical clustering of the genes differentially regulated in H2B R95A mutant under no treatment or rapamycin treatment as compared to H2B WT.** This analysis is based on a subset of n=73 genes (rows) which were found to be differentially expressed in H2B R95A mutant. Hierarchical clustering generated a tree (dendrogram) and group similar genes together. The color-ratio bar at the top indicates intensity of gene up-regulation (red), down-regulation (green) and no change (black).


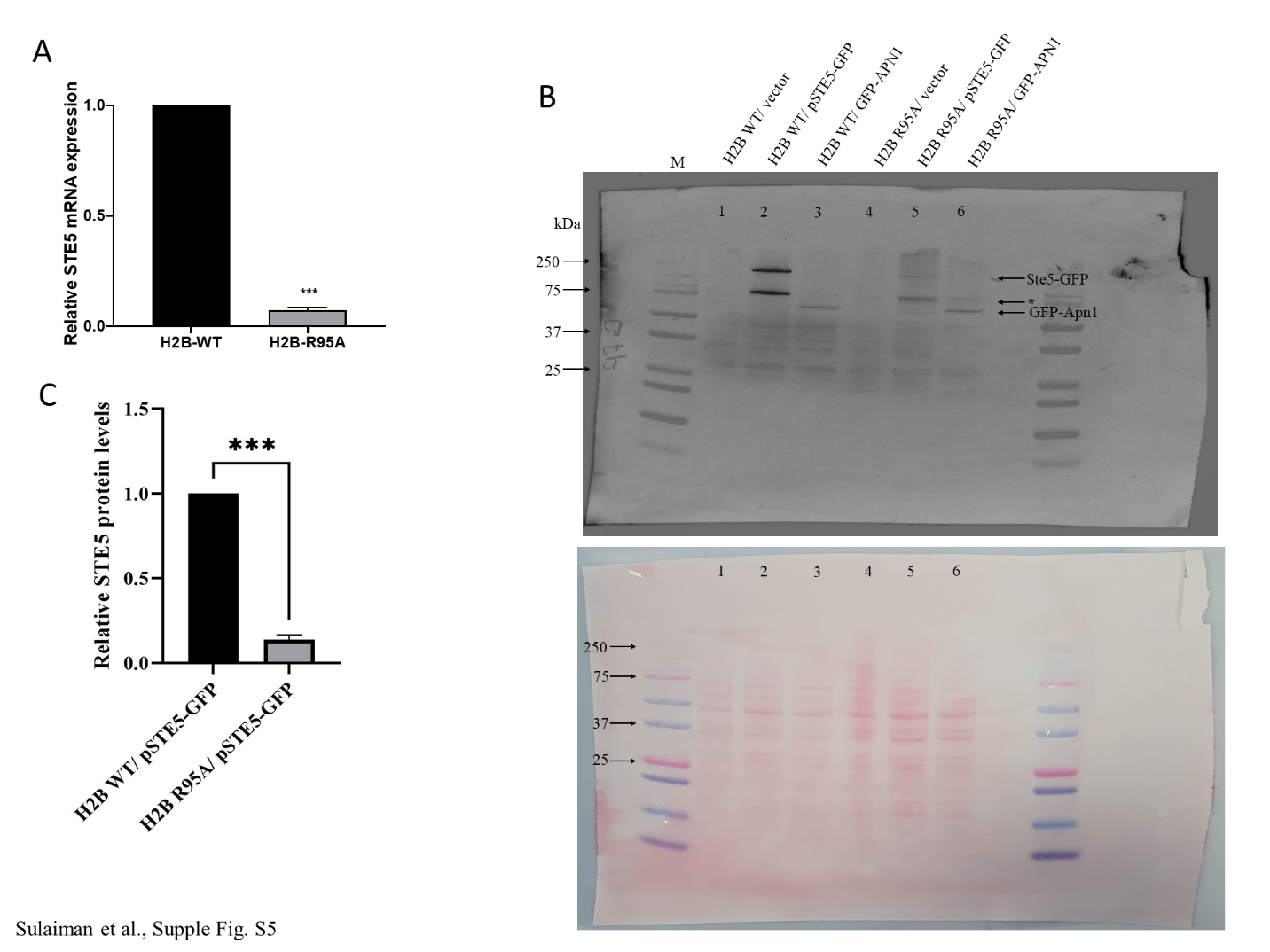


**Figure S5: H2B R95A mutant drastically reduces the expression of the *STE5* gene and its encoded protein. A,** Relative mRNA expression of *STE5* gene by qPCR analysis. Total RNA was isolated from the H2B WT and H2B R95A strains and 0.5 µg was used for cDNA preparation, subjected to qPCR analysis (see Materials and Methods). The *ACT1* gene was used as a control. The data are representative of four biological replicates and analyzed by student *t*-test. *** is equivalent to P-value < 0.001. **B**, Immunoblot analysis of Ste5-GFP and GFP-Apn1 expression in the H2B WT and H2B R95A strains. The blot was probed with anti-GFP antibodies (upper panel). The lower panel was stained with Ponceau to monitor for equal protein loading. M, prestained protein markers in kDa. Arrows indicate the position of the GFP-tagged proteins, and the asterisk indicates a fragmented species of Ste5-GFP. **C**, The expression level of the full-length Ste5-GFP in the H2B R95A mutant was quantified from panel B and expressed relative to the level detected in the H2B WT strain.

**
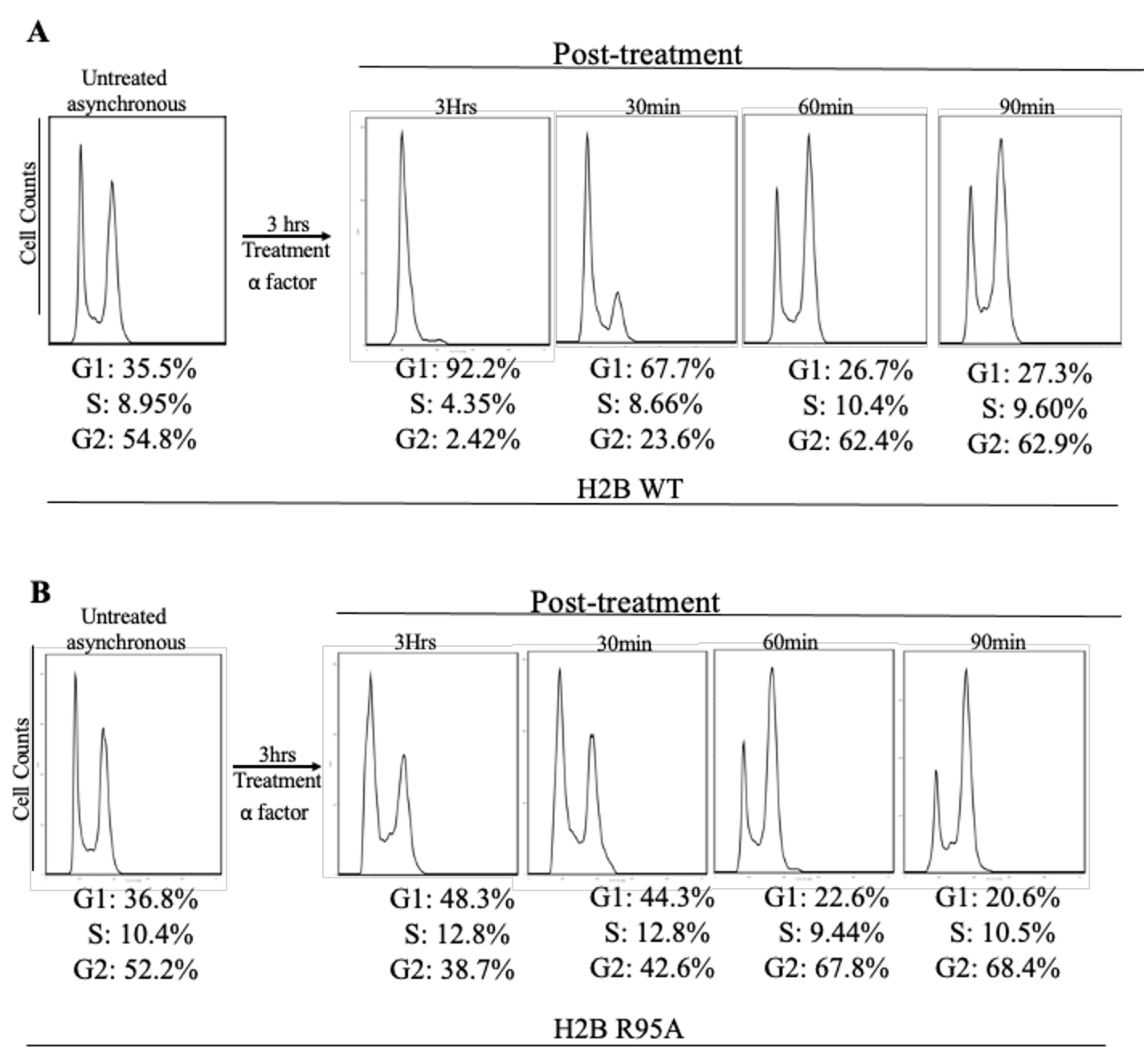
**

**Figure S6: α-factor induces G_1_ cell cycle arrest in the H2B WT, but not in the H2B R95A mutant strain**. **A and B**, Briefly, overnight cells were sub-cultured for 3 hours and samples were taken for asynchronous growth followed by treatment with α-factor (4 µg/ml for 3 hours). Cells were washed free of the α-factor, released into fresh media and samples taken at the indicated times were processed using FACS analysis and the image treated by the Flowjo software. The results are representative of two independent analyses.

**
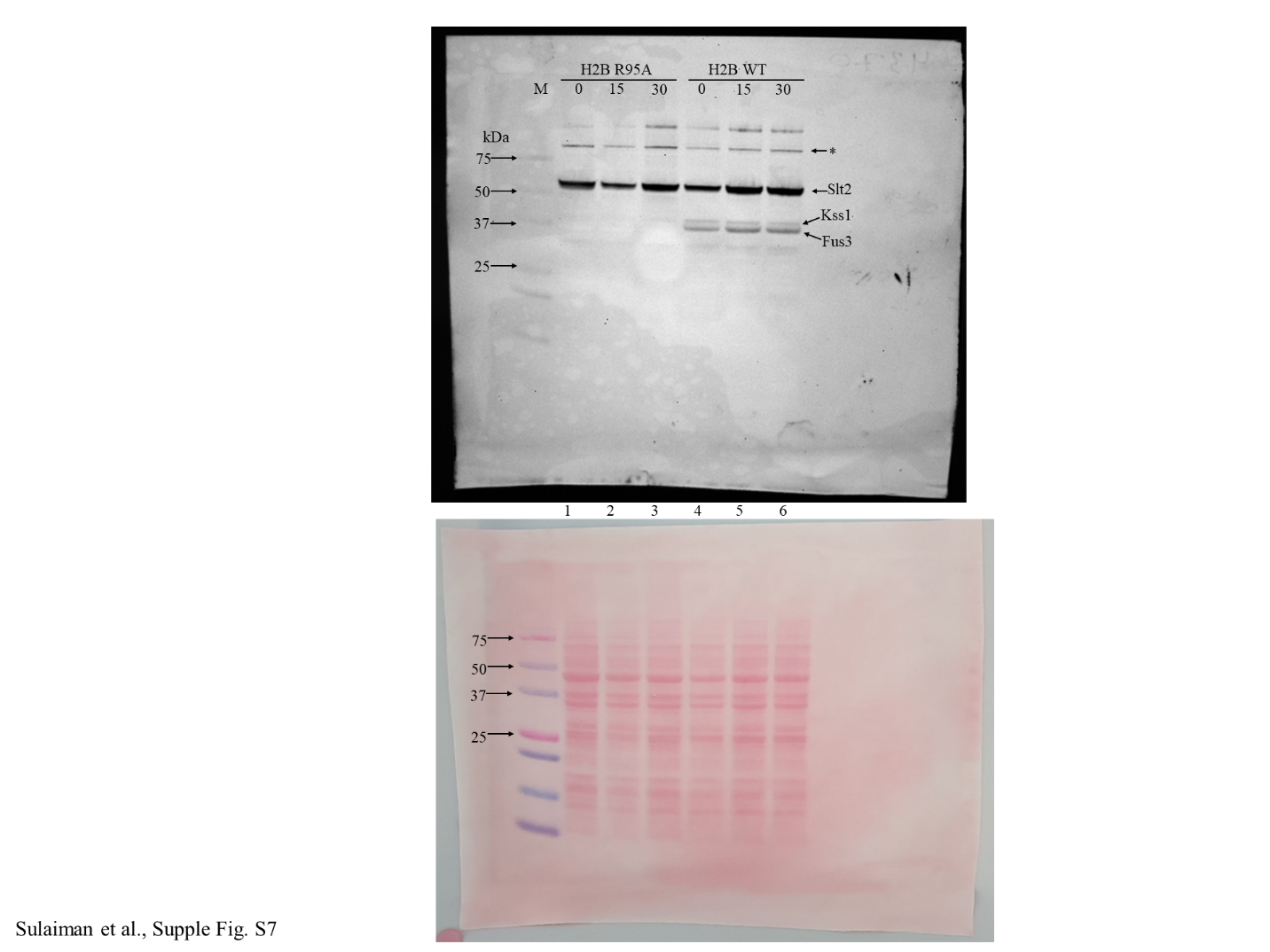
**

**Figure S7: The MAP kinase Fus3, a downstream component of the pheromone pathway, is phosphorylated in the H2B WT, but not detected in the H2B R95A mutant.** Exponentially growing cells were treated without (time zero) and with rapamycin (200 ng/ml). Samples were taken at 15 and 30 min for total protein extraction by trichloroacetic acid (TCA). The TCA extracted proteins were analyzed by immunoblot and probed with anti Erk1/2 to detect the MAPKs (see Materials and Methods). The antibody detects three know proteins in yeast, the Slt2 kinase, Kss1, and Fus3. The asterisk denotes a high molecular weight polypeptide of unknown origin. The lower panel was stained with Ponceau to monitor for equal protein loading from the TCA samples. M, prestained protein markers in kDa.

**
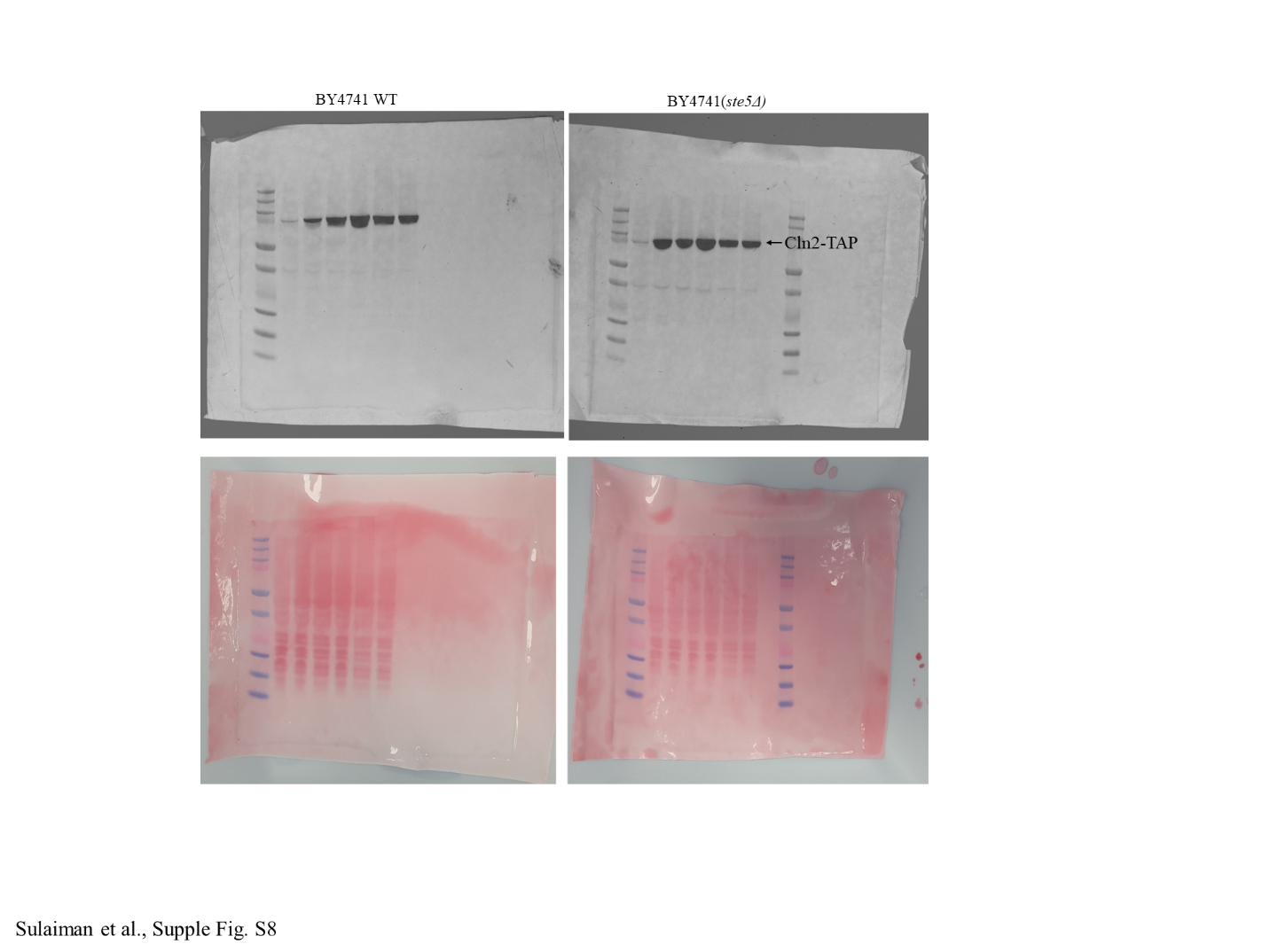
**

**Figure S8.** **The full immunoblot shows that rapamycin treatment causes rapid accumulation of cyclin Cln2 and at a higher level in the *ste5Δ* mutant, as compared to the WT.** Exponentially growing cells from strain BY4741 (WT) carrying the TAP tag at the endogenous *CLN2* gene locus and the isogenic strain deleted for the *STE5* gene were treated without (time zero) and with rapamycin (200 ng/ml). Samples were taken at 30, 60, 120, 180, and 240 mins for total protein extraction by TCA. The TCA extracted proteins were analyzed by immunoblot and probed with anti-TAP, which recognizes the protein A domain of the Cln2-TAP-tagged fusion protein (see Materials and Methods). The lower panel was stained with Ponceau to monitor for equal protein loading from the TCA samples. M, prestained protein markers in kDa.

**
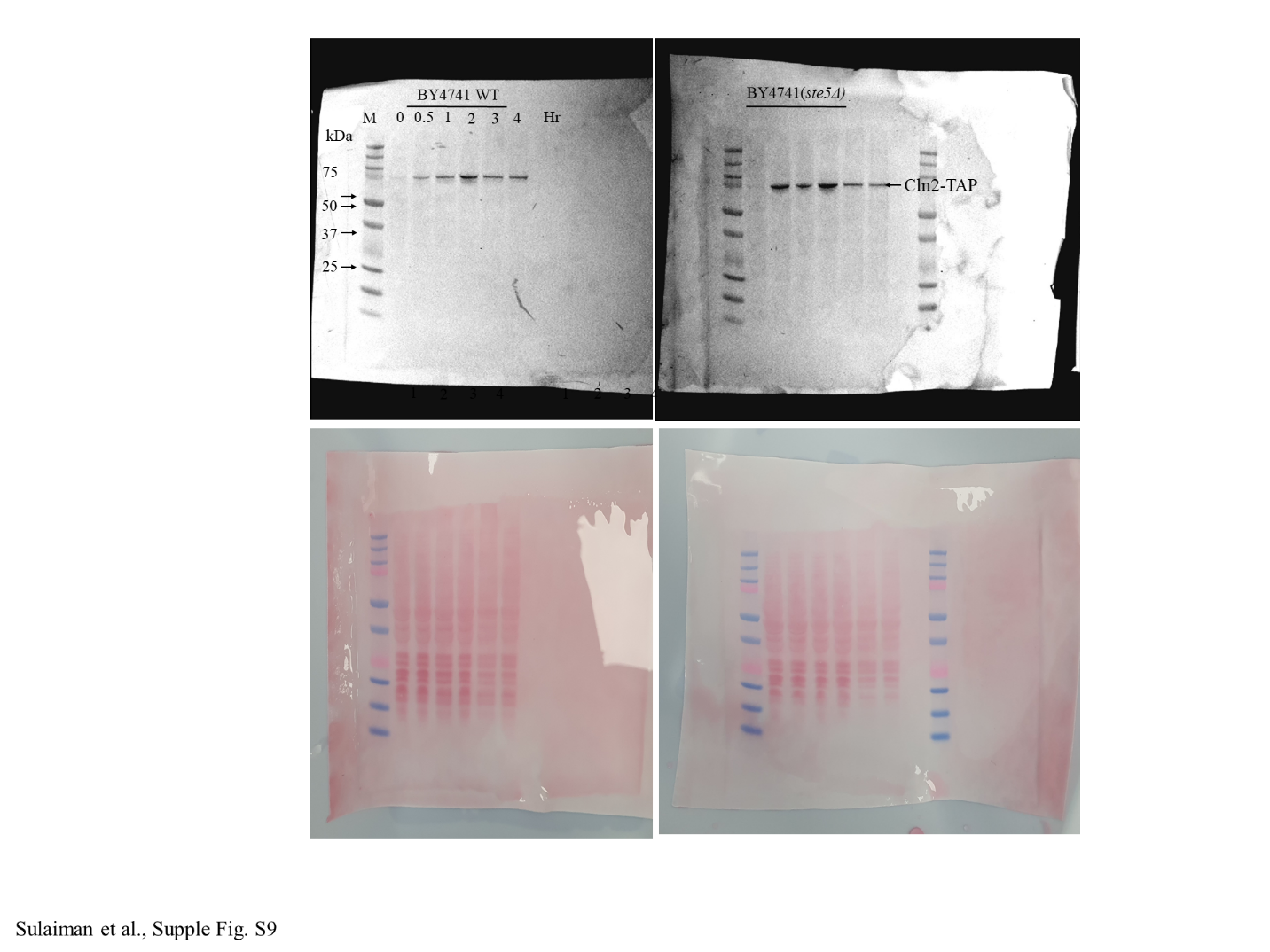
**

**Figure S9.** **This immunoblot is identical to that shown in Supplemental Fig. S8, except displayed at a reduced exposure.**

**
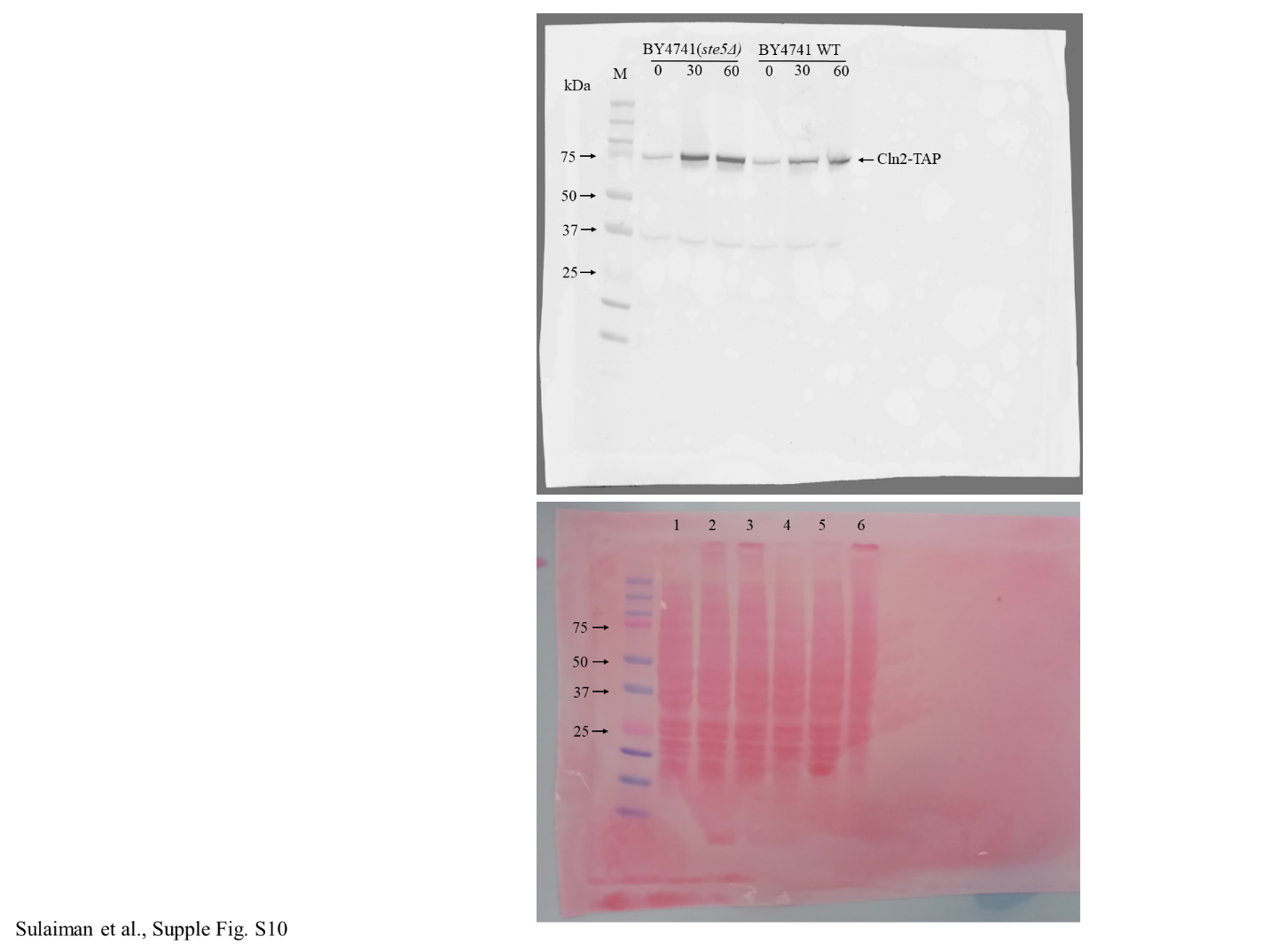
**

**Figure S10:**  **An independent repeat showing that rapamycin treatment causes rapid accumulation of cyclin Cln2 and at a higher level in the *ste5Δ* mutant, as compared to the WT.** The experiment was conducted as in Supplemental Fig. S8, except the cells were untreated and treated with rapamycin (200 ng/ml) for 30 and 60 mins.


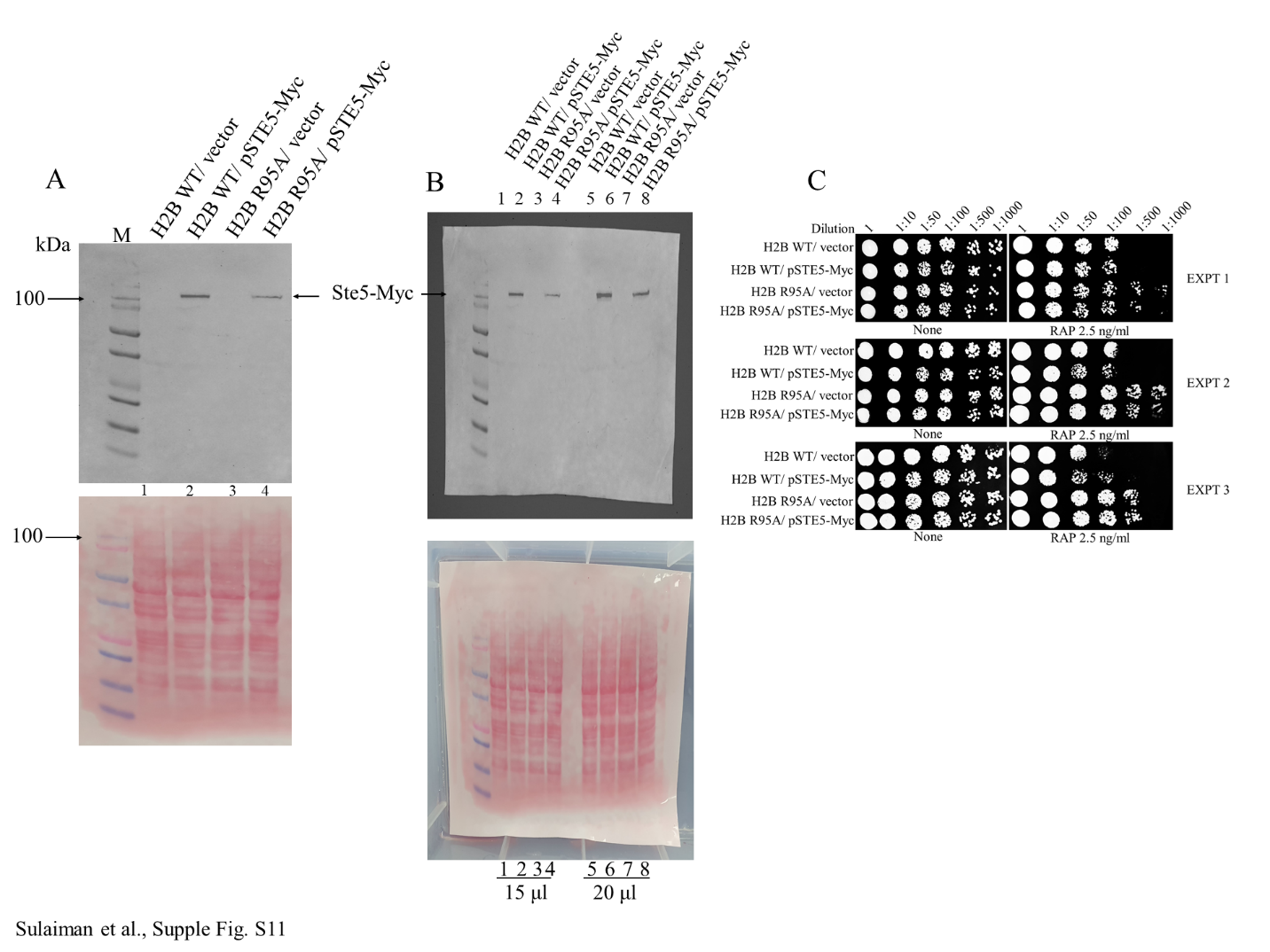


**Figure S11.** **Ste5-MYC expression and sensitization of the H2B WT and the H2B R95A strains to rapamycin**. **A**, Immunoblot showing Ste5-MYC expression level from the constitutive promoter *ADH*. Analysis was done with proteins obtained by TCA extraction. **B**, Full blot as in **A**, except showing two different amounts (15 and 20 µl) of proteins loaded on the gel. **C,** Spot test analysis of the indicated strains was performed as described in Fig. 1A. The spot tests were from three independent biological replicates and the plates were photographed at 48 hrs.
